# Stable Gastric Pentadecapeptide BPC 157 as Useful Cytoprotective Peptide Therapy in the Heart Disturbances, Myocardial Infarction, Heart Failure, Pulmonary Hypertension, Arrhythmias, and Thrombosis Presentation

**DOI:** 10.3390/biomedicines10112696

**Published:** 2022-10-25

**Authors:** Predrag Sikiric, Mario Udovicic, Ivan Barisic, Diana Balenovic, Gordana Zivanovic Posilovic, Dean Strinic, Sandra Uzun, Suncana Sikiric, Ivan Krezic, Helena Zizek, Haidi Yago, Slaven Gojkovic, Ivan Maria Smoday, Luka Kalogjera, Hrvoje Vranes, Marija Sola, Sanja Strbe, Antun Koprivanac, Ivica Premuzic Mestrovic, Tomislav Mestrovic, Predrag Pavic, Anita Skrtic, Alenka Boban Blagaic, Martina Lovric Bencic, Sven Seiwerth

**Affiliations:** 1 Department of Pharmacology, School of Medicine, University of Zagreb, 10000 Zagreb, Croatia; 2 Department of Internal Medicine, School of Medicine, University of Zagreb, 10000 Zagreb, Croatia; 3 Department of Pathology, School of Medicine, University of Zagreb, 10000 Zagreb, Croatia; 4 Department of Surgery, School of Medicine, University of Zagreb, 10000 Zagreb, Croatia

**Keywords:** stable gastric pentadecapeptide BPC 157, peptide therapy, hearth disturbances, myocardial infarction, arrhythmias, congestive heart failure, pulmonary hypertension, thrombosis

## Abstract

In heart disturbances, stable gastric pentadecapeptide BPC 157 especial therapy effects combine the therapy of myocardial infarction, heart failure, pulmonary hypertension arrhythmias, and thrombosis prevention and reversal. The shared therapy effect occurred as part of its even larger cytoprotection (cardioprotection) therapy effect (direct epithelial cell protection; direct endothelium cell protection) that BPC 157 exerts as a novel cytoprotection mediator, which is native and stable in human gastric juice, as well as easily applicable. Accordingly, there is interaction with many molecular pathways, combining maintained endothelium function and maintained thrombocytes function, which counteracted thrombocytopenia in rats that underwent major vessel occlusion and deep vein thrombosis and counteracted thrombosis in all vascular studies; the coagulation pathways were not affected. These appeared as having modulatory effects on NO-system (NO-release, NOS-inhibition, NO-over-stimulation all affected), controlling vasomotor tone and the activation of the Src-Caveolin-1-eNOS pathway and modulatory effects on the prostaglandins system (BPC 157 counteracted NSAIDs toxicity, counteracted bleeding, thrombocytopenia, and in particular, leaky gut syndrome). As an essential novelty noted in the vascular studies, there was the activation of the collateral pathways. This might be the upgrading of the minor vessel to take over the function of the disabled major vessel, competing with and counteracting the Virchow triad circumstances devastatingly present, making possible the recruitment of collateral blood vessels, compensating vessel occlusion and reestablishing the blood flow or bypassing the occluded or ruptured vessel. As a part of the counteraction of the severe vessel and multiorgan failure syndrome, counteracted were the brain, lung, liver, kidney, gastrointestinal lesions, and in particular, the counteraction of the heart arrhythmias and infarction.

## 1. Introduction

Numerous key clinical trials published or presented at major international conferences over the course of 2021 were reviewed as the most valuable contributions to clinical cardiology (for review, see, i.e., [1]). Heart failure data focused on trials with sodium–glucose cotransporter 2 (SGLT2) inhibitors, sacubitril/valsartan, and mavacamten for hypertrophic cardiomyopathy [1]. Proprotein convertase subtilisin/kexin type 9 (PCSK9) inhibitors were centered in the prevention trials [1].

On the other hand, as a new attempt, from the cytoprotection viewpoint and potential involvement of the cytoprotective agents, we reviewed the potential significance in the heart disturbances of the therapy with the stable gastric pentadecapeptide BPC 157 (for review, see, i.e., [2,3,4,5,6,7,8,9]). It appeared, as a peptide native and stable in human gastric juice, as a late outbreak of the cytoprotection/organoprotection concept of Robert and Szabo, a concept mostly from the stomach studies [10,11,12,13,14,15,16,17] for epithelial and endothelial protection, like the previous theoretical/practical breakthroughs in the 1980s and brain–gut axis and gut–brain axis (for review, see, i.e., [2,3,4,5,6,7,8]). However, its compelling basic highlights (particular vascular effect, activation of the collateral pathways) [18,19,20,21,22,23,24,25,26,27,28,29,30,31,32,33,34,35,36,37,38,39,40,41] in the most valuable animal models might still be far from the vast clinical evidence obtained in the huge number of clinical trials [1]. Nevertheless, it might challenge further therapy use. 

The novel point, the particular vascular effect for the epithelial and endothelial protection, activation of the collateral pathways [18,19,20,21,22,23,24,25,26,27,28,29,30,31,32,33,34,35,36,37,38,39,40,41] might arise from the original BPC 157 cytoprotective evidence [2,3,4,5,6,7,8]. Given that the conceptual Robert and Szabo’s stomach/cytoprotection relation is translated to the protection of other tissues (Robert and Szabo’s organoprotection), BPC 157, as a peptidergic agent that is native and lacking degradation in the human gastric juice and is stable for more than 24 h, conceptually emerges as a novel cytoprotection mediator with particular cytoprotective capabilities, which are effectively translated into pleiotropic beneficial effects [2,3,4,5,6,7,8]. With selective effect on both epithelial and even more endothelial tissue damage (maintaining both epithelial and endothelial integrity), it was very safe, and there were no side effects in clinical trials (i.e., used in ulcerative colitis); a lethal dose (LD1) was not achieved in toxicology studies [2,3,4,5,6,7,8]. Thus, in general terms, given its easy applicability (including via a therapeutic per-oral regimen), BPC 157 therapy leads to the upgraded minor vessel taking over the function of the failed major vessel to compensate and reestablish the reorganized blood flow [18,19,20,21,22,23,24,25,26,27,28,29,30,31,32,33,34,35,36,37,38,39,40,41], which occurs as the recovery of endothelium function [2,3,4,5,6,7,8]. Thereby, with BPC 157 “bypassing key”, there were reported the prevention and the reversal of the myocardial infarction (for review, see, i.e., [19,24,27,29,31,37,38,39,40]), arrhythmias (for review, see, i.e., [19,22,23,24,27,28,29,31,37,38,39,40,41]), and from different origins, heart failure [19,24,27,29,31,37,38,39,40,41] and pulmonary hypertension [41] and thrombosis [18,19,23,24,27,28,29,31,37,38,39,40]. Together, these might be the compelling evidence for the implemented concept of cytoprotection (i.e., the process by which chemical compounds provide protection to cells against harmful agents) [2,3,4,5,6,7,8]. 

Furthermore, in Robert and Szabo’s original view [10,11,12], the realization of the cytoprotection/organoprotection concept benefits from the pleiotropic beneficial effects of the cytoprotection agents. Thus, more tissues are protected with better cytoprotective activity. Moreover, conceptually, cell protection per se precludes simultaneous adverse effects on other tissues as well. This wide cytoprotection agenda might be distinctively from a highly focused background, such as the concept of glucose toxicity of the SGLT2 inhibitors [42,43,44]. It should be noted that the prototype SGLT1 and SGLT2 blocker, phorizin, did not achieve suitability for human use due to considerable problems (i.e., diarrhea, dehydration, and malabsorption due to small intestine SGLT1 inhibition) [42,43,44]. Possibly, this cytoprotection approach [2,3,4,5,6,7,8] might not limit the current therapy of heart failure SGLT2 inhibitors [42,43,44]. Ketoacidosis, urosepsis, pyelonephritis, acute kidney injury, anaphylaxis, and angioedema appeared as additional adverse effects of canagliflozin and empagliflozin use [42,43,44]. Moreover, it was pointed out that gliflozin-induced infection, cancers, liver injury, hypoglycemia, and hypovolemia dehydration, hypovolemia, but mostly hypotension or orthostatic hypotension, and hemoconcentration (and thereby, the risk for thrombosis) affected bone metabolism and increased the risk of fracture [42,43,44]. Likewise, the wide cytoprotection agenda might also be distinctive from the focused background of the angiotensin-converting-enzyme inhibitors (ACE inhibitors), angiotensin II receptor blockers [45,46,47], or beta-blockers [48].

Thus, there is hope that this particular vascular recovering effect of the BPC 157 therapy [18,19,20,21,22,23,24,25,26,27,28,29,30,31,32,33,34,35,36,37,38,39,40,41] could finally bring into practice the huge theoretical importance of cytoprotective agents (i.e., selectivity for the damaged epithelium and/or selectivity for the damaged endothelium [10,11,12]) long ago proposed in the series of cytoprotection funding reports [10,11,12,13,14,15,16,17]. In the case of stable gastric pentadecapeptide BPC 157, the selectivity for both damaged epithelium and damaged endothelium might rapidly activate cytoprotection maxim endothelium maintenance → epithelium maintenance, making BPC 157 “bypassing key” capable of realizing all therapy aspects of the cytoprotection concept [2,3,4,5,6,7,8]. Furthermore, as a cytoprotection advantage, with this agenda (i.e., making possible the recruitment of collateral blood vessels, compensating vessel occlusion, and re-establishment of blood flow or bypassing the occluded or ruptured vessel), the stable gastric pentadecapeptide BPC 157/heart disturbances issue (for review, see, i.e., [2,3,4,5,6,7,8]) implied the innate resolving of the commonly presented Virchow [8] and wide beneficial therapeutic effects. Thereby, there was a therapeutic resolution of the severe syndromes, including vascular and multiorgan failure otherwise inducing following vessel(s) occlusion (arteries [18,19,20], veins [22,23,24,25,26,27], arteries and veins [28,29,30], peripherally and/or centrally), and other alike procedures [31,32,33,34,35,36,37] and damaging agents [38,39,40,41]. A large pathology that commonly appeared was resolved [19,24,27,29,31,37,38,39,40]. The intra-cranial (superior sagittal sinus), portal and caval hypertension, and aortal hypotension were resolved [19,24,27,29,31,37,38,39,40]. The severe lesions in the brain, heart (congestion and endocardial infarction), lung, liver, kidney, and gastrointestinal tract were counteracted [19,24,27,29,31,37,38,39,40]. Major vessels congestion (i.e., inferior caval vein, superior mesenteric vein) was reversed to normal vessel presentation, and the failure of the collapsed azygos vein presented as the reactivated pathway for blood flow direct delivery [19,24,27,29,31,37,38,39,40]. The ECG disturbances [19,24,27,29,31,37,38,39,40] were resolved. Otherwise, overwhelming arterial and venous thrombosis, peripherally and centrally, was almost annihilated as part of the resolved stasis [19,24,27,29,31,37,38,39,40]. Thereby, there was a selective effect on the damaged endothelium, depending on the injured vessel injury and rapid recruitment of the appropriately activated collaterals [19,24,27,29,31,37,38,39,40] (i.e., in rat, azygos vein, in addition to providing direct blood flow delivery, i.e., [37,38,39,40], resembles the atrial myocardium [49]) as a useful peptide therapy given the BPC 157 “bypassing key”. This might rapidly occur upon its application [19,24,27,29,31,37,38,39,40] (note, endothelium recovery is known to occur with cytoprotection agents within less than 1 min in stomach injury studies [12]).

Thereby, from this particular point of view, we will focus on the potential significance of the stable gastric pentadecapeptide BPC 157 in heart disturbances therapy. Possibly, this therapy might equally include the myocardial infarction (for review, see, i.e., [19,24,27,29,31,37,38,39,40]), arrhythmias (for review, see, i.e., [19,22,23,24,27,28,29,31,37,38,39,40,41]), acute and chronic heart failure [19,24,27,29,31,37,38,39,40,41] and pulmonary hypertension [41] and thrombosis [18,19,23,24,27,28,29,31,37,38,39,40], prevention and reversal, all as interrelated and closely connected effects.

Particular consideration may be in the interaction with many molecular pathways [3,4,5,6,20,22,50,51,52,53,54,55,56,57], taking as evidence the BPC 157/NO-system’s particular importance (i.e., the endothelium and thrombocytes function both maintained (for review, see, i.e., [2,3,4,5,6,7,8])). BPC 157 therapy counteracted thrombocytopenia in rats underwent major vessel occlusion and deep vein thrombosis [22] and counteracted thrombosis in all vascular studies [18,19,23,24,27,28,29,31,37,38,39,40]), and coagulation pathways not affected [58,59,60]. Further arguments might be controlling vasomotor tone and the activation of the Src-Caveolin-1-eNOS pathway [53,54]. This likely occurred as the particular modulatory effects on the nitric oxide (NO)-system as a whole, induced NO-release on its own [61,62,63], counteracted NO-synthase (NOS)-inhibition [61] (i.e., N(G)-nitro-L-arginine methylester (L-NAME)-hypertension and pro-thrombotic effect) [58,62], and counteracted NO-over-stimulation [61] (L-arginine-hypotension and anti-thrombotic, pro-bleeding effect) [58,62]. Likewise, the isoprenaline myocardial infarction was counteracted by NO-effect [38]. Furthermore, due to its close interaction with NO-system, as NO acts as an endogenous cardioprotectant antifibrillatory factor [64,65] and BPC 157 has no proarrhytmic effect by itself, BPC 157 might counteract various arrhythmias, including those aggravated by NOS-blockade [66,67,68,69,70,71,72] (note, first communication was about the shortened duration of arrhythmias during hypoxia and the reoxygenation period in isolated guinea pig hearts [73]). Moreover, there might be modulatory effects on the prostaglandins system [2,3,4,5,6,7,8,74]; BPC 157 counteracted NSAIDs toxicity (associated with the occurrence of symptoms of heart failure [75,76], including prolonged bleeding and thrombocytopenia [58,59,60] (for review, see, i.e., [74]) and indomethacin-induced leaky gut syndrome, in particular (for review, see, i.e., [5])).

### 1.1. Cytoprotection Background (Direct Epithelial Cell Protection) for BPC 157 Beneficial Activity

The wide applicability of the original postulates of Robert and Szabo’s cytoprotection concept (for review, see, i.e., [10,11,12,13,14,15,16,17]) might approach the entire problem of heart failure. This wide approach might be useful as a large number of the concomitant diseases with heart failure might be the key for the therapeutic effects [77,78,79], as the stable gastric pentadecapeptide BPC 157 pleiotropic effect belongs to the cytoprotective class of agents (for review, see, i.e., [2,3,4,5,6,7,8]). Note the general background of the BPC 157 beneficial effects on various organs injuries (for review, see, i.e., [2,3,4,5,6,7,8]), which helps one recognize the wide significance of the cytoprotection concept of Robert’s (direct epithelial cell protection) [10] and Szabo’s (direct endothelium cell protection) [12] that is initiated in the stomach to be further generalized. The foundation of the cytoprotective agents’ putative activities in the stomach studies was the initial basic point for their possible therapy extension [10,11,12,13,14,15,16,17]. In general, BPC 157 successfully follows the common cytoprotective principle: the original cytoprotective agent with a prime beneficial effect in the stomach (direct (epithelial) cell protection) had to be transmitted to similar beneficial effect in other organ lesions as well (cytoprotection → organoprotection) [10,11,12,13,14,15,16,17] (for review, see, i.e., [2,3,4,5,6,7,8]). Noteworthily, BPC 157 therapy, in practical terms (native and stable in human gastric juice for more than 24 h, and, thereby, easily applicable), unlike standard cytoprotective agents, fully presumes original cytoprotective requirements (for review, see, i.e., [2,3,4,5,6,7,8]). Thereby, the extent of the obtained beneficial effects largely overrides the range of the beneficial effects commonly reported with the standard cytoprotective agents (for review, see, i.e., [2,3,4,5,6,7,8]) (i.e., prostaglandins’ beneficial effects on stomach [10], intestine [13], liver [80], pancreas [13], kidney [81], and heart [82]). Unlike the effectiveness only given before injury (prophylactic effect) of the standard cytoprotective agents (for review, see, i.e., [10,11]), BPC 157, in addition to its prophylactic effect, has a strong curative effect given even much later after injury induction, during ischemia as well as during reperfusion (for review, see, i.e., [2,3,4,5,6,7,8]). Illustratively, as mentioned before, in the vascular studies, as a part of the severe vascular and multiorgan failure syndrome counteraction, there was counteraction of the brain, heart, lung, liver, kidney, and gastrointestinal lesions [18,19,23,24,27,28,29,31,37,38,39,40]. Moreover, in other separate studies, there was counteraction of the brain [83], spinal cord [35,36], heart failure [84], lung [41,85,86,87], liver lesions [88,89,90], liver, gastrointestinal and brain lesions [91,92,93,94,95,96], and kidney [97,98,99] and pancreas [100,101] lesions. There was also a strong wound-healing effect (for review, see, i.e., [3,102]). Thereby, there was the curing of the skin [53,55,103,104,105], nerve [106], tendon [50,51,107,108,109,110,111], muscle [110,111,112,113,114,115], ligament [116], and bone [117,118,119] injuries that spontaneously might not heal. In particular, there was a capability to simultaneously organize the healing of the different tissues (as an example occurred the healing of the osteotendinous junction [108,109] and the healing of the myotendinous junction [111] (and neuromuscular junction function recovering [68]) or the healing of the fistulas, external and internal [120]). Likewise, in particular regard for wounding [3,102], these realized healing effects in the various wounds [53,55,103,104,105,106,107,108,109,110,111,112,113,114,115,116,117,118,119,120] might evidence the realized healing process after blood vessel are ruptured as a whole, and thereby, as we claimed [59], a distinctive effect on all four major events in clot formation and dissolution was fully accomplished. This meant a highly utilizable special effect, especially with heart failure therapy [18,19,23,24,27,28,29,31,37,38,39,40]. Moreover, BPC 157 is very safe, with no adverse effect in clinical trials (i.e., ulcerative colitis), and lethal dose (LD1) was not achieved in toxicology studies (for review, see, i.e., [2,3,4,5,6,7,8]). 

Thereby, these beneficial effects (for review, see, i.e., [2,3,4,5,6,7,8]) fulfill the cytoprotection (organoprotection) frame at the general level (implied direct cell protection) [10,11,12,13,14,15,16,17], with all of the mentioned beneficial effects as pre-requests for the resolved heart disturbances. In these terms, the effect on the heart (cardioprotection) might be an additional part of the cytoprotective activity (for review, see, i.e., [10,11,12,13,14,15,16,17,121,122,123]), and, in particular, it might be commonly taken as proof and consequence of its innate cytoprotective activity (for review, see, i.e., [2,3,4,5,6,7,8]). In addition to being native and stable in human gastric juice for more than 24 h, BPC 157 was found in situ hybridization and immunostaining studies in humans to be largely distributed in tissues [3,102] and may have additional physiologic regulatory roles [8,102] as it is thought to be a novel cytoprotective mediator. Furthermore, there is a particular healing effect depending on the tissue involved (for review, see, i.e., [3,102]). Particularly, there is an improved healing effect (for review, see, i.e., [3,102]) for eye injuries (no angiogenesis) [124] versus advanced angiogenesis in other tissues (i.e., tendon, muscle) [110] (for review, see, i.e., [3,102]), which together might provide evidence that BPC 157s beneficial effect is even more complex and tissue specific. Illustratively, BPC 157 eye drops successfully closed perforating corneal incisions in rats; controls developed new vessels that grew from the limbus to the penetrated area, whereas BPC 157-treated rats generally had no new vessels, and those that did form in the limbus did not make contact with the penetrated area [124]. Thus, important for heart healing as well, BPC 157 certainly might control one of the most important aspects of the cytoprotection and cytoprotective agents activity in long terms (i.e., days): the angiogenesis (corneal avascularity as “angiogenic privilege”) (for review, see, i.e., [3,102]).

### 1.2. Cytoprotection Background (Direct Endothelial Cell Protection) for BPC 157 Beneficial Activity

Overwhelmingly focused on stomach cytoprotection, the pioneers, Robert (direct epithelial cell protection) [10] and Szabo (direct endothelium cell protection) [11], estimated in stomach damage studies the maxim endothelium maintenance → epithelium maintenance as rapid injury, rapid defensive response, vascular injury within less than 1 min, thrombus and stasis [11], thereby, although not claimed, Virchow triad circumstances. Moreover, finally, the rapid recovery of damaged endothelium occurred as a shared effect of the cytoprotective agents within stomach cytoprotection [11]. With BPC 157 effect (see above), there is an advanced practical realization of the original maxim functioning [8]. This might be the rapid upgrading of the minor vessel to take over the function of the disabled major vessel [18,19,23,24,27,28,29,31,37,38,39,40], as the particular effect on the vessel relied on the given injury. Furthermore, this implies competing with the Virchow triad circumstances devastatingly present, making possible the recruitment of collateral blood vessels, compensating vessel occlusion, and reestablishing blood flow or bypassing the occluded or ruptured vessel [18,19,23,24,27,28,29,31,37,38,39,40]. Illustrative examples might be the therapy of glaucoma in rats after the cauterization of three of the four episcleral veins [26], venous congestion, and the increased intraocular pressure and consequent glaucoma injurious course [26]. For the BPC 157 therapy importance estimation [18,19,23,24,27,28,29,31,37,38,39,40], one remaining episcleral vein was upgraded so that BPC 157 therapy did compensate all functions; otherwise, inescapable venous congestion and the increased intraocular pressure and consequent glaucoma injurious course fully reversed [26]. Moreover, BPC 157 therapy (the rapid upgrading of the collateral pathways) has cured many severe syndromes, including multiorgan and vascular failure [18,19,23,24,27,28,29,31,37,38,39,40], and heart dysfunction and thrombosis as cause–consequence, in particular. Otherwise, without therapy, these syndromes were commonly presented in rats with the permanent occlusion of major vessels (veins and/or arteries [18,19,20,22,23,24,25,26,27,28,29,30], peripherally and centrally), major intoxication (lithium, alcohol) [39,40], acute pancreatitis [37], myocardial infarction [37], and maintained intra-abdominal hypertension [31]. Its applicability in the rapid upgrading of the collateral pathways may likely provide an additional beneficial effect for the heart functions, and various vessel tributaries, and normalization/attenuation of the intracranial (sinus sagittal) hypertension, portal and caval hypertension and aortal hypotension, and counteraction of the multiorgan failure syndrome [18,19,23,24,27,28,29,31,37,38,39,40].

We suggested these particular effects and this background as a network of the evidence for the physiologic significance of the revealed BPC 157/vascular-system interplay [18,19,20,21,22,23,24,25,26,27,28,29,30,31,32,33,34,35,36,37,38,39,40,41] (i.e., in situ hybridization and immunostaining studies in humans evidenced BPC 157 large distribution in tissues [102] and suggested its additional physiologic regulatory roles [8,102]). 

In this agenda, we will further review heart disturbances and specifically indicate the particular effects of BPC 157 therapy.

## 2. Myocardial Infarction

### 2.1. Isoprenaline Myocardial Infarction

Myocardial infarction induced with the suited doses of isoprenaline and re-infarction (after two isoprenaline applications) [38] and reversed with the stable gastric pentadecapeptide BPC 157 (for review, see, i.e., [2,3,4,5,6,7,8]) may represent the usefulness of the peptide therapy (Table 1). In consideration of the myocardial infarction, arrhythmias, heart failure, pulmonary hypertension, and thrombosis presentation, the therapy of myocardial infarction might occur as definitive proof of the successful outcome. Isoprenaline myocardial infarction was used as the first prototype of rapid methods in rats, verified to fairly mimic acute myocardial infarction in humans [125]. In addition, there is the additional therapy target, early vascular failure, recently pointed out [18,19,23,24,27,28,29,31,37,38,39,40], and the upgrading of the minor vessel to take over the function of the disable major vessel, competing with the Virchow triad circumstances devastatingly present, making possible the recruitment of collateral blood vessels also in isoprenaline rats [38]. Therefore, important for the isoprenaline myocardial infarction, and generally, we revealed these antecedent early noxious effects [18,19,23,24,27,28,29,31,37,38,39,40], and the early vascular failure as being isoprenaline-induced, which is, so far, less recognized, and less considered, peripherally and centrally. Centrally, without therapy, in the isoprenaline rats, there was intracranial (superior sagittal sinus) hypertension, severe brain swelling, large intracerebral hemorrhage, and intraventricular hemorrhage in the third ventricle, marked karyopyknosis in the cerebral, cerebellar cortex and hippocampus, while the hypothalamus appeared to be relatively spared (only rare karyopyknotic cells) [38]. Peripherally, there was portal and caval hypertension; aortal hypotension; congested (i.e., inferior caval vein and superior mesenteric vein) and failed (azygos vein) blood vessels; multiple organ lesions, i.e., the heart dilatation, myocardial congestion and confluent areas of subendocardial ischemic myocytes, ECG disturbances (i.e., giant T-wave); and severe congestion in the lung, liver, kidney, and gastrointestinal tract [38]. Venous and arterial thrombosis were progressing peripherally and centrally [38]. Essentially (i.e., providing common vascular disability point and heart dysfunction), this corresponds to the described large syndrome commonly seen with the endothelium damaging agent overdose, alcohol [40] and lithium [39], acute pancreatitis [37] and maintained intra-abdominal hypertension, grade III and grade IV [31], as well as corresponding to the described occlusion syndrome with major vessels occlusion, peripheral [19,24,29] or central [27]. Thus, these disturbances, and consequently, the beneficial counteraction by BPC 157 therapy, may have a general significance [38]. Commonly, these disturbances [38], presented as shared occlusion-like and occlusion syndromes [19,24,27,29,31,37,38,39,40], may provide additional prototypes for the heart lesions, vascular failure, and therapy possibilities. Of note, all of these disturbances were consistently attenuated with BPC 157 therapy application and the activation of the collateral pathways, relayed on the given injury [18,19,20,21,22,23,24,25,26,27,28,29,30,31,32,33,34,35,36,37,38,39,40,41]. 

Thus, given the initial infarct induction and re-infarction (the myocardial lesions after two isoprenaline applications) and that BPC 157 markedly counteracts myocardial isoprenaline lesions, the findings provide multidirectional evidence [38]. Specifically, there is a large range of the BPC 157 regimens (ng-µg) [38] and huge range of the therapy possibilities (i.e., a sustained effect given before isoprenaline, and a rapid effect given after isoprenaline, mortality absent in BPC 157 rats) [38]. Thus, there is consistent and quite complete evidence (i.e., reduction in all of the routine necrosis markers, grossly no visible infarcted area, attenuated histological damage, ECG (no ST-T ischemic changes), and echocardiography (preservation of systolic left ventricular function) damage and oxidative stress parameters decreased) [38]. The interaction with eNOS and COX2 gene expression, and counteraction of the aggravation effect of the NOS-blocker, L-NAME [38], might suggest that NO system function might be accordingly recovered.

The given therapy effect on the initial heart infarct induction and re-infarction [38], as well as indicated anti-thrombotic [18,19,23,24,27,28,29,31,37,38,39,40] and anti-arrhythmic effect [19,22,23,24,27,28,29,31,37,38,39,40,41]) of the BPC 157 therapy, might strongly support the comparable BPC 157 therapy effect on stroke in rats and therapy in the reperfusion after bilateral clamping of the common carotid arteries for a 20-min period [20]. As assessed at 24 h and 72 h of the reperfusion, the therapy counteracted both early and delayed neural hippocampal damage, achieving full functional recovery (Morris water maze test, inclined beam-walking test, lateral push test) [20]. mRNA expression studies at 1 and 24 hr, provided, in the hippocampus, strongly elevated (Egr1, Akt1, Kras, Src, Foxo, Srf, Vegfr2, Nos3, and Nos1) and decreased (Nos2, Nfkb) gene expression (Mapk1 not activated), as a way how BPC 157 may act [20]. Considering the ischemic event itself in rats with the occluded superior sagittal sinus, without therapy, the complete infarction was within 24 h and marked karyopyknosis at 48 h [27]. In all BPC 157 rats, the consistent neuroprotective effect appeared in all brain areas, and there were only a few karyopyknotic neurons [27]. 

In this, the BPC 157 therapy prompt activation of the azygos vein, as before upon BPC 157 therapy [19,22,23,24,27,28,29,31,37,38,39,40]) goes along with the combining notation about the atrial myocardium and azygos vein resemblance in the rat [49]. Consequently, the counteraction of the myocardial infarction course might go via azygos vein activation that BPC 157 therapy might promptly initiate (note, without BPC 157 therapy, the azygos vein remained completely collapsed, in the isoprenaline-treated rats, in particular) [38]. There might be prompt direct delivery of blood flow, compensation, and no more failed vessel (i.e., initially congested inferior caval vein and superior mesenteric vein recovered to the normal vein presentation), and blood flow reorganized, seeable with the absent caval and portal hypertension and the decreased brain swelling, and the decreased intracranial (superior sagittal sinus) hypertension [30]. The restored function immediately upon administration might regain reversal of the venous and intracranial hypertension [38] since recovered heart function [38] might ascertain the ability to drain venous blood adequately for a given cerebral blood inflow without raising venous pressures. Grossly, the counteracted progressing venous and arterial thrombosis, peripherally and centrally, counteracted ECG disturbances, and no heart dilatation and absent gastrointestinal (stomach) lesion [38] were present with all microscopic findings. The myocardial presentation was without congestion, and confluent areas of subendocardial ischemic myocytes and the lung, liver, kidney, and gastrointestinal tract were with only mild congestion. Intracerebral hemorrhage and intraventricular hemorrhage in the third ventricle were absent, and karyopyknosis was almost annihilated in all of the brain areas. As mentioned for isoprenaline-myocardial infarction [38], the same chain of events might be seen as a shared principle [19,22,23,24,27,28,29,31,37,38,39,40]. Illustratively, the rats underwent the endothelium damaging agent overdose, alcohol [40] and lithium [39], bile duct ligation, acute pancreatitis [37] and maintained intra-abdominal hypertension, grade III and grade IV [31], as well as the described occlusion syndrome with major vessels occlusion, peripheral [19,24,29] or central [27].

Thus, this might be recognized as a special heart-brain-blood vessels interacting axis (providing in the counteracted multiorgan failure, the attenuated myocardial lesions, and arrhythmias as an achieved important tool, either as a cause or as a consequence), peripheral and central interplay, as an essential defensive response [19,22,23,24,27,28,29,31,37,38,39,40]. This would be further analyzed specifically with respect to the particular myocardial lesion and heart failure in the large syndrome commonly seen also with the endothelium damaging agent overdose, alcohol [40] and lithium [39], acute pancreatitis [37], and maintained intra-abdominal hypertension, grade III and IV [31], as well as in the described occlusion syndrome with major vessels occlusion, peripheral [19,24,29] or central [27].

It should be noted that to verify the suggested peripheral and central interplay, the studies also implied several routes of BPC 157 application, with equipotent beneficial therapy effect [19,22,23,24,27,28,29,31,37,38,39,40]. Local application at the swollen brain implies a direct effect; intraperitoneal or intragastric administrations mean a systemic effect; µg- and ng-regimens mean a common beneficial effect [27]. Conceptually, intragastric application benefits BPC 157 importance as an original cytoprotective anti-ulcer peptide (i.e., epithelium, endothelium maintenance and protection) [2,3,4,5,6,7,8].

### 2.2. Heart Failure

As a follow-up of the described therapy course in the rats with acute myocardial infarction [38], we evidenced that BPC 157 therapy might counteract acute heart failure as it might compensate for the effect of the severe blood flow restriction to the heart (Table 1). This might occur in a very short time with the major vessel occlusion [19,24,27,29], artery [19] or vein [24], or artery and vein [29], peripherally and centrally, and trapped large blood volume [19,24,29]. The evidenced marked congestion within the myocardium and large coronary branches included the occlusion of the superior mesenteric vein [24], the occlusion of the superior mesenteric artery [19], the simultaneous occlusion of both the superior mesenteric artery and the superior mesenteric vein [29], and centrally, the occlusion of the superior sagittal sinus [27]. The subendocardial infarct, as well as congestion within the myocardium, regularly appeared in the rats with the occluded superior mesenteric vein and artery [29].

Furthermore, as mentioned before (see Section 2.1, Isoprenaline-myocardial infarction [38]), we demonstrated that, in addition to the isoprenaline-myocardial infarction [38], the other major noxious events [31,37,38,39,40] also acutely caused the prominent heart failure and widespread dysfunction similar to that observed in rats after the occlusion of the peripheral [19,24,29] and central [27] vessels. These were the endothelium damaging agents, i.e., alcohol [32] or lithium [31], bile duct occlusion (acute pancreatitis) [29], organs and vessels compression (intra-abdominal hypertension) [31]. As emphasized before, these occlusion-like syndromes [19,24,27,29] shared the previously described peripheral and central deficits noted in the occlusion syndromes and were largely counteracted by the given BPC 157 therapy. Characteristically, with intragastric absolute alcohol, a prototype noxious agent in the cytoprotection studies, there was timely advancing heart failure [40]. There were rapidly produced heart dilatation and lesions worsening (i.e., 1 min < 5 min < 15 min < 30 min; moderate congestion ˂ tissue congestion and persistent hemorrhage < passive congestion in the myocardium, with acute subendocardial infarct (note contribution of low aortic pressure) < prominent congestion and acute subendocardial infarct) [40]. Rats treated with lithium sulfate overdose for three days since the beginning of treatment presented with severe myocardial congestion, along with subendocardial infarcts and neutrophilic infiltration of the infarcted areas, in particular after the second and third doses of lithium [39]. In the rats with a ligated bile duct, heart dilatation and marked myocardial congestion was consistently noted at 30 min, 5 h, and 24 h ligation time [37]. Already within a very short time with severe intra-abdominal hypertension 25 mmHg, grade III, there was myocardial congestion and sub-endocardial infarction, which appeared as the ultimate outcome [31]. The myocardial congestion and sub-endocardial infarction occurred in an even shorter time with the more severe intra-abdominal hypertension, grade IV (i.e., 30 mmHg/30 min, 40 mmHg/30 min, 50 mmHg/25 min) [31]. Rats with the occluded major vessel(s) superior mesenteric vessels or superior sagittal sinus commonly presented prominent congestion and acute subendocardial infarct (rats with the occluded superior mesenteric artery and vein) [19,24,27,29]. 

On the other hand, commonly, BPC 157 therapy counteracted heart failure, and the BPC 157 rats exhibited either normal heart microscopic presentation or markedly attenuated lesions [19,24,27,29]. With normal heart microscopic presentation of the rats, they were found with occluded major vessels [19,24,27,29], or challenged with intragastric absolute alcohol [40], an overdose of lithium [39], or maintained severe intra-abdominal hypertension, grade III and grade IV [31]. Likewise, with BPC 157, in the rats with the occluded bile duct, the early regimen resulted in no changes in 30 min ligation time and only mild myocardial congestion at 5 h and 24 h ligation time [37]. Thus, it might be that the countermeasures commonly achieved with BPC 157 therapy might ascertain the normal heart presentation despite the continuous presentation of the severely harmful circumstances of the major vessel(s) occlusion, bile duct occlusion, severe intra-abdominal hypertension, or noxious agents’ application [19,24,27,29,31,37,38,39,40]. Evidently, as mentioned before, the bypassing loops were reliant on the corresponding injurious occlusion and might reestablish the reorganized blood flow, thus compensating vessel occlusion and markedly attenuating the harmful syndrome severity [19,24,27,29,31,37,38,39,40]. There, the number of vessels was identified as useful collateral. There were the left ovarian vein, inferior mesenteric vein, inferior anterior pancreaticoduodenal vein, superior anterior pancreaticoduodenal vein, pyloric vein, (para)sagittal venous collateral circulation, and azygos vein as venous pathways, the inferior mesenteric artery and inferior anterior pancreaticoduodenal artery as alternative arterial pathways (i.e., occluded superior mesenteric artery) [18,19,23,24,27,28,29,31,37,38,39,40]. Together, these might clearly suggest that this BPC 157 therapy effect might have common application [18,19,23,24,27,28,29,31,37,38,39,40]. 

It might be claimed that this therapeutic effect might also ascertain the resolution of the concomitant arrhythmias in heart failure (in heart failure, arrhythmias are commonly acknowledged as the final cause of death [126]). Commonly, there was marked tachycardia, prolonged PQ and QTc intervals, and ST elevation (major vessel(s) occlusion, bile duct occlusion, alcohol intoxication) as identifiers of acute thrombotic coronary occlusion and right heart failure [18,19,23,24,27,28,29,31,37,38,39,40]. These all rapidly disappeared under all the BPC 157 regimens (as also noticed in the Pringle maneuver ischemia, reperfusion, portal triad temporary occlusion, and in the Budd–Chiari syndrome with BPC 157 therapy in rats) [18,19,23,24,27,28,29,31,37,38,39,40]. Note, it might be a biventricular failure that was accordingly counteracted (as a follow-up of the right heart failure, there was marked congestion of the inferior caval vein, superior mesenteric vein, liver, kidney, and gastrointestinal tract while congested lung with hemorrhage also evidenced left heart failure, all counteracted by BPC 157 therapy) [18,19,23,24,27,28,29,31,37,38,39,40]. Lithium overload induced rapidly significant ST elevation, prolonged QTc intervals, and atrioventricular conduction disturbances (i.e., total AV block), in addition to marked bradycardia [39]. Contrarily, BPC 157-treated rats exhibited no repolarization changes, and they showed the conduction system of the heart functioned normally and the normal heart frequency at all time checkpoints and none of the atrioventricular conduction disturbances [39]. There might be additional particular relevance. Namely, the used regimen (500 mg/kg, ip, once daily for three consecutive days) (i.e., higher than usual lithium regimens [127,128,129] but markedly below usual LD50 for lithium application in rats [130]) might be closer to those used in patients considering the conversion of animal doses to human-equivalent doses based on body surface area [131]. Maintaining the severe intra-abdominal hypertension 30 mmHg/30 min, 40 mmHg/30 min, and 50 mmHg/25 min revealed the downhill course timely along with the nodal rhythm, with dominant ST-elevation and bradycardia [31]. Extreme bradycardia and asystole appeared as the ultimate outcome [31]. Contrarily, all BPC 157-treated rats exhibited consistently preserved heart function, with fewer ECG disturbances [31] (preserved sinus rhythm, with occasional first-degree AV block, without ST-elevation, extreme bradycardia and asystole) [31] and normal heart microscopic presentation. To emphasize the achieved therapy effect significance, we should stress the persisting worst circumstances of intra-abdominal hypertension, grade III and grade IV [31], pushing up the diaphragm, the most constrained thoracic cavity [31], the rapid transmission of the increased pressure between the three body cavities [31]. 

It might be claimed that this therapy recovery effect in heart failure might also ascertain the resolution of the concomitant thrombosis in heart failure [18,19,23,24,27,28,29,31,37,38,39,40]. In all of the experiments, the progressing thrombosis in the veins and arteries, peripherally and centrally (i.e., as noted in the inferior caval vein, portal vein, lienal vein, superior mesenteric vein, superior sagittal sinus, abdominal aorta, hepatic artery, superior mesenteric artery) was almost annihilated [18,19,23,24,27,28,29,31,37,38,39,40]. Therefore, along with recovered heart function and annihilated arrhythmias, resolved thrombosis might be the final identifier of the resolved stasis as well, and removed Virchow triad circumstances [18,19,23,24,27,28,29,31,37,38,39,40].

Considering the more extended experiments, there was by BPC 157 therapy the counteraction of the monocrotaline-induced pulmonary hypertension in rats [41]. It might be that BPC 157 therapy might affect the monocrotaline course as a whole since pulmonary damage (hours), edema (after one week), pulmonary artery hypertension (after two weeks), right ventricle hypertrophy (after three weeks), and considerable fatal rate (after four weeks), given the same beneficial effect of both the prophylactic regimen and the delayed therapeutic regimen [41]. Analyzing how pentadecapeptide BPC 157 prevents and counteracts monocrotaline-induced pulmonary arterial hypertension and cor pulmonale in rats, the evidence seems to be quite compelling. The experimental protocol [41] comparable to the clinical situation [132] (pulmonary hypertension in the untreated monocrotaline group on day 14) meant the delayed therapy initiation (i.e., day 14) was well-chosen [133] (avoiding the misleading considering the therapeutic effect [132] with shorter premature intervals (day 11 or day 12) after monocrotaline [132,134]). Additionally, the range of the assessed disturbed parameters (which were all counteracted) fully corresponded to other studies [132]. They included disturbed wall thickness, total vessel area, and heart frequency; QRS axis deviation; QT interval prolongation (known to correlate with pulmonary pressure and right ventricle dilation and inversely correlate with right ventricle function [135]; right ventricle hypertrophy; increased right ventricle weight [136,137]; an increase in right ventricle systolic pressure; mortality; and bodyweights loss). In particular, the reduced body weight as a marker of clinical deterioration in the animal, as in the patient, again accords with previous studies [136]. Likewise, with respect to the timing of the initiation of therapy being crucial [132], there was the prophylactic effect (just after monocrotaline), pulmonary hypertension not even developed, as well as the therapeutic effect (on day 14 after monocrotaline) the advanced pulmonary hypertension was rapidly attenuated and then completely eliminated (delayed regimen) [41]. Thus, there is compelling evidence [41] that the right ventricle can be therapeutically targeted in pulmonary arterial hypertension [135].

Further extension toward the chronic heart failure effect [84] was based on the estimated role of the endothelin, and thereby NO-system [137], doxorubicin model [138], and delayed BPC 157 therapy application [84]. After the doxorubicin regimen (total dose of 15 mg/kg intraperitoneally, divided at six time points, every third day for 14 days to induce congestive heart failure), and after four weeks of rest, assessed in mice and rats with advanced disease, the increased big endothelin-1 (BET-1) and plasma enzyme levels (CK, MBCK, LDH, AST, ALT), before and after the subsequent 14 days of therapy, and clinical status (hypotension, increased heart rate, and respiratory rate, and ascites) every two days [84]. Without therapy, throughout 14 days, both rats and mice further raised BET-1 serum values and aggravated clinical status, while enzyme values maintained their initial increase [84]. BPC 157 (10 µg/kg) and amlodipine treatment reversed the increased BET-1 (rats, mice), AST, ALT, CK (rats, mice), and LDH (mice) values. BPC 157 (10 ng/kg) and losartan opposed further increase of BET-1 (rats, mice). Losartan reduces AST, ALT, CK, and LDH serum values. BPC 157 (10 ng/kg) reduces AST and ALT serum values. The clinical status of chronic heart failure in rats and in mice is accordingly improved by the BPC 157 regimens and amlodipine [84]. However, indicatively, in translation to the counteracted hypotension, no dyspnea with increased heart and respiratory occurred in BPC 157 treated animals, whereas hypotension and dyspnea with increased heart rate and respiratory rate persisted in the losartan and amlodipine treated animals [84].

Thus, BPC 157 therapy as applied as intragastric application, per-oral in drinking water, might exert the reversal of the doxorubicin-induced congestive heart failure, effective even in the advanced status of failing heart in rats and mice studied by the reversal of the BET-1 plasma level [84]. These findings’ relevance goes with the known local activation of the big endothelin-1 (BET-1) system [139,140,141,142]. This characterizes most cardiovascular diseases (including doxorubicin-congestive heart failure [84]), renal failure, and functional and structural changes in the cardiovascular system [142,143,144,145,146,147,148,149,150]. Commonly, endothelin relationship with NO-system dysfunction and BET-1 plasma levels might well recognize the particular BPC 157/NO-system relation and BPC 157/vascular system interplay [18,19,20,21,22,23,24,25,26,27,28,29,30,31,32,33,34,35,36,37,38,39,40,41] along with its innate cytoprotection background (for review, see, i.e., [2,3,4,5,6,7,8]) on the severity of congestive heart failure [150,151,152,153,154], and the effects of therapy as well as the rate of ET-1 synthesis [143,144,152,153,154]. Note, BPC 157 therapy with counteracted heart lesions might actually improve the effectiveness of drugs used in chemotherapy for cancer patients, both solid tumors and leukemia, anthracyclines, i.e., doxorubicin, epirubicin, and daunorubicin, otherwise markedly limited with damage to the heart [155,156,157].

Moreover, particularly with respect to doxorubicin-heart lesions, BPC 157 may be more than one of a large number of the cardioprotective agents (for review, see, i.e., [155]) and might particularly consider the special vulnerability of the heart to injury from free radicals, and lower level of protective enzymes such as superoxide dismutase [158,159]. Namely, the evidence that BPC 157 may have a particular effect on the heart [19,24,27,29,31,37,38,39,40,41,84] goes with its acting as free radical scavenger [5,6], counteraction of the free radicals-induced lesions in different tissues [5,6,19,22,24,25,27,28,29,30,32,33,34,38,39,90,160,161], and thereby, due to its particular cytoprotective/cardioprotective activity [2,3,4,5,6,7,8], it might beneficially affect the myocardial lesions, in particular [19,24,27,29,31,37,38,39,40,41,84]. As an additional advantage, BPC 157 itself also showed a prominent anti-tumor effect [6,162] and might counteract the VEGF-tumor-promoting effect [163], as well as tumor cachexia [6]. Thus, its cardioprotective intervention during anthracycline therapy should be without reducing the anti-tumor efficacy and likely due to its pleiotropic beneficial effect [for review see, i.e., [2,3,4,5,6,7,8]), it should also decrease negative effects on toxicities other than cardiac damage (i.e., BPC 157 reduced cyclophosphamide-induced gastric and duodenal lesion, and bladder toxicity [160,161]).

### 2.3. Heart Failure Concomitant Pathology

As mentioned before in the acute myocardial infarction studies [19,24,27,29,31,37,38,39,40,41] and BPC 157 therapy application, the evidence that the heart failure cause–consequence occurred with the wide range of the concomitant multiorgan failure and initiated multicausal noxious circuit that might also be counteracted by BPC 157 therapy, was specifically elaborated. This was conducted in a series of the different major noxious events and BPC 157 therapy effects, peripherally or centrally, and/or peripherally and centrally [19,24,27,29,31,37,38,39,40,41]. As challenges confronted with BPC 157 therapy beneficial effects, we used distinctive noxious procedures. This was a confrontation with the occlusion of the superior mesenteric vein, the occlusion of the superior mesenteric artery, the occlusion of the superior mesenteric vein and artery, the occlusion of the superior sagittal sinus, the intragastric application of the absolute alcohol, subsequent intraperitoneal administrations of the lithium overdose, and maintained severe intra-abdominal hypertension grade III and grade IV [19,24,27,29,31,37,38,39,40,41]. Thereby, there was a large range of the therapy effect, given the similar heart failure described before, and all concomitant similar to multiorgan failure, relayed to the various noxious conditions (i.e., constant major vessel(s) occlusion, constant mechanical compression of the organs and vessels, abrupt challenge of the intragastric bolus (absolute alcohol) or repeated subsequent applications of the lithium-overdose) [19,24,27,29,31,37,38,39,40,41]. These multiple organ failure lesions might be perceived as the lesions following diverse noxious agents’ direct effect, highlighted in the cytoprotection studies, and vice versa; the counteraction, pleiotropic beneficial effect by BPC 157 therapy might also be understood in the general cytoprotection terms [2,3,4,5,6,7,8].

Centrally, without therapy, there was intracranial (superior sagittal sinus) hypertension as a shared disturbance (note, the regular negative values (i.e., −27 mmHg) changed to the high positive values), quickly counteracted by BPC 157 therapy (reestablished negative pressure values) [27]. Thus, given the recovered heart failure, there was a recovered ability to drain venous blood adequately for a given cerebral blood inflow without raising venous pressure (in contrast, the harmful inability suddenly causes venous and intracranial hypertension) [27]. Moreover, the instant severe brain swelling was shared (i.e., in lithium-rats [39] as well as in the alcohol-rats [40]; the brain volume proportional with the change in the brain surface area revealed an immediate increase to 120% of the healthy presentation). Likewise, there was also shared therapy effect of BPC 157 therapy, and promptly attenuated brain swelling. Regularly, without therapy, all investigated noxious procedures (i.e., alcohol intoxication, lithium intoxication, maintained severe intra-abdominal hypertension, vessels occlusion, superior mesenteric artery and/or vein, and superior sagittal sinus) [19,24,27,29,31,37,38,39,40] presented the severely damaged brain areas, i.e., cerebral and cerebellar cortex, hypothalamus/thalamus, and hippocampus. Prominent edema and large areas with increased numbers of karyopyknotic cells occurred as shared harmful and inevitable outcomes. These were all attenuated or even eliminated by BPC 157 therapy. As a distinctive point, there was a large intracerebral hemorrhage fully counteracted by BPC 157 therapy. Illustratively, the lithium rats exhibited hemorrhage in the deeper brain, both gray and white matter. Rats with bile duct occlusion presented pronounced intracerebral hemorrhage affecting large areas of the corpus callosum, amygdala, thalamus, neocortex, and striatum, and intraventricular hemorrhage in the third and lateral ventricles [39]. After intragastric alcohol, brain edema after 1 and 5 min, with vascular congestion progressed after 15 and 30 min to generalized congestion, edema, and intracerebral hemorrhage, with degenerative changes in the cerebral and cerebellar neurons indicating toxic changes created by the ethanol [40]. In the rats with occluded superior mesenteric artery and superior mesenteric vein [29], there was a subarachnoid hemorrhage at the base of the brain in the cerebellar area, and more karyopyknotic cells in the cerebral and cerebellar cortex, hippocampus, and hypothalamus/thalamus. In the rats with the occluded bile duct, there were timely progressing pronounced intracerebral hemorrhages in areas of the corpus callosum, amygdala, thalamus, neocortex, and striatum, intraventricular hemorrhage involving the third and lateral ventricles and more karyopyknotic cells in the cerebral and cerebellar cortex, hippocampus, and hypothalamus/thalamus [37]. In the rats with the occluded superior sagittal sinus, complete infarction appeared at 24 h and marked karyopyknosis at 48 h [27]. Rats with maintained severe intra-abdominal hypertension exhibited subarachnoid hemorrhage at the base of the brain in the cerebellar area and more karyopyknotic cells in the cerebral and cerebellar cortex, hippocampus, and hypothalamus/thalamus [31]. These brain lesions appeared to be distinctively affected by high intra-abdominal pressure; i.e., the most progressive hippocampal neuronal damage was found with the highest intra-abdominal pressure [31].

Thus, the indication of the brain lesions and hemorrhage and their counteraction evidence might be seen as particular maxim (i.e., occluded superior mesenteric vessels vs. occluded superior sagittal sinus vs. alcohol/lithium application vs. bile duct occlusion vs. maintained intra-abdominal pressure, prime peripheral lesions vs. prime central lesions vs. peripheral and central lesions) [19,24,27,29,31,37,38,39,40]. This might suggest that the heart failure cause–consequence might occur in a bidirectional way that might be both beneficially affected by the BPC 157 therapy.

At the periphery, there were portal and caval hypertension and aortal hypotension, markedly attenuated or even eliminated by BPC 157 therapy [19,24,27,29,31,37,38,39,40]. In particular, depending on the prime injurious challenge (i.e., vascular occlusion vs. bile duct occlusion vs. intragastric absolute alcohol vs. intraperitoneal lithium challenge vs. maintained intra-abdominal hypertension), the lesion shared considerable severity along with the described heart failure and prominent brain lesions [19,24,27,29,31,37,38,39,40]. These were all attenuated or even eliminated by BPC 157 therapy [19,24,27,29,31,37,38,39,40]. Illustratively, rats with maintained severe intra-abdominal hypertension exhibited lung parenchyma with marked congestion and large areas of intra-alveolar hemorrhage, vascular dilation of the liver parenchyma, and severe congestion of renal tissue [31]. With a severity increase from the upper toward the lower part of the gastrointestinal tract, there was transmural hyperemia of the entire gastrointestinal tract, stomach, duodenum, and small and large bowel wall, along with a reduction in the villi in the intestinal mucosa, crypt reduction with focal denudation of superficial epithelia, and dilatation of the large bowel [31]. Likewise, without therapy, all of the rats with the occluded superior mesenteric vessel(s) (i.e., occluded superior mesenteric artery, or occluded superior mesenteric vein, or occluded both superior mesenteric artery and vein) exhibited marked transmural congestion in an ascending sequence from the stomach to the large bowel [19,24,29]. This might be the particular maxim: the stomach (dilated capillaries in the lamina propria) < duodenum (mild mucosal injury, blunt villi, and mild hyperplasia of the crypts) < small bowel wall (focal hemorrhage in the lamina propria) < large bowel wall (severe mucosal injury with lumen dilatation and reduction of crypts). There were the focal thickening of the alveolar membranes, lung congestion, pulmonary edema, intra-alveolar hemorrhage, focal interstitial neutrophil infiltration, mild activation of Kupffer cells, and severe enlargement of sinusoids with liver congestion, mild degeneration of proximal tubules, severe renal vascular congestion and interstitial edema [19,24,29]. A similar presentation appeared with the central occlusion [27]. In rats with the occluded superior sagittal sinus, the marked congestion in the heart tissue within the myocardium and large coronary branches exhibited huge additional pathology [27]. There were the gross stomach lesions, and microscopically, erosive gastritis, the liver congestion, and lung congestion with intra-alveolar hemorrhage and pyknotic hepatocyte nuclei, hyaline tubular cylinders, cell degeneration of proximal and distal tubule with cytoplasmic vacuolization in the kidney after both a short-term (15 min ligation time, and period thereafter) and a long-term (24 h, 48 h) period. In the rats challenged with the intragastric absolute alcohol instillation [40], the large gross hemorrhagic lesions and severe pathology in the stomach (i.e., mucosal surface erosion, even in the macroscopically intact areas) were along since very early time (i.e., 1 min post-alcohol) with the progression of the other lesions. They exhibited lung tissue congestion with persistent hemorrhage, liver lesions, congestion, and a ballooning of hepatocytes in zone three of the liver lobules and kidney lesions, congestion, and its progression in the renal tissues presented with dilated and congested small, medium, and large blood vessels, as well as glomeruli. In the rats challenged with the subsequent lithium overdose, along with the described heart failure, there was the progressing lesions presentation [39]. They exhibited in the lungs marked congestion, intra-alveolar hemorrhage, and interstitial neutrophil infiltration, liver with congestion and dilatation of central veins, sinusoids, and portal tracts vessels, marked congestion and vacuolization of the renal tubular epithelia with degenerative changes and marked congestion in the gastrointestinal tract (and gross stomach lesions). As an interesting point, severe muscular weakness might appear immediately, while a decrease in muscular fibers microscopically appears later. In the rats with the occluded bile duct, there was timely progress of a large range of lesions [37]. They exhibited marked lung parenchyma congestion along with intra-alveolar hemorrhage. Moreover, they had marked dilatation and congestion of blood vessels in the portal tracts, central veins, and sinusoids, along with the zones of confluent necrosis affecting the liver lobuli and the portal tract. There was marked dilatation and congestion of blood vessels in the kidney tissue as well as glomeruli and marked congestion in the gastrointestinal tract (and gross stomach and duodenal lesions). These were along with the timely progressing acute pancreatitis lesions. They exhibited grossly separate to confluent hemorrhagic zones and/or foci of necrosis, and microscopically to diffuse edema of interlobar septe, interlobular septe, interacinal spaces, diffuse expansion of intercellular spaces, increased number of necrotic acinar cells/HPF (extensive confluent necrosis) and foci of hemorrhage and fat necrosis, perivascular increased infiltration leukocytes/HPF, and confluent microabscesses [37].

Thus, the indication of the larger range of peripheral lesions and hemorrhage and their counteraction evidence might be seen as a particular and complex maxim. This complex maxim might be the particularity of the prime lesion (i.e., occluded superior mesenteric vessels vs. occluded superior sagittal sinus). This complex maxim equally supposed one prime lesion (alcohol-hemorrhagic lesions, bile duct occlusion pancreatitis) or many prime lesions (lithium application, maintained intra-abdominal pressure), and thereby, prime peripheral lesions vs. prime central lesions vs. peripheral and central lesions. This might suggest that the heart failure cause–consequence might occur in the periphery between the heart and affected organ (i.e., lung, liver, kidney, gastrointestinal tract) in a multidirectional way that might be all beneficially affected by the BPC 157 therapy.

As mentioned before, peripherally and centrally, there was progressing thrombosis in the vein and artery [18,19,23,24,27,28,29,31,37,38,39,40]. As emphasized before, the cloths were assessed in the inferior caval vein, portal vein, lienal vein, superior mesenteric vein, superior sagittal sinus, abdominal aorta, hepatic artery, and superior mesenteric artery. Thereby, the heart failure, the consistent large concomitant congestion multiorgan pathology, and widespread thrombosis cause–consequence relation might be the final identifier of the overspread stasis as well, and the overwhelming Virchow triad circumstances that therapy might counteract [18,19,23,24,27,28,29,31,37,38,39,40].

Thereby, it might be that the alcohol intoxication [40], lithium intoxication [39], maintained severe intra-abdominal hypertension [31], and vessels occlusion, superior mesenteric artery and/or vein, and superior sagittal sinus [19,24,27,29] appeared as a multiple occlusion syndrome that could not be avoided unless therapy was given. Regularly, reciprocal changes in the abdominal, thoracic, and brain cavities rapidly transmitted through the venous system rapidly appeared as determinants of vascular failure. Therefore, with BPC 157, there might be a rapid improvement of venous system function as an essential common point to prevent and reverse the noxious chain of events and attenuate all harmful consequences. For illustration, an activated azygos vein as a rescuing pathway, avoiding both the lung and liver, combines the inferior caval vein and superior caval vein via direct blood delivery [19,24,27,29,31,37,38,39,40]. Thus, an activated azygos vein shunt could reorganize blood flow and instantly attenuate the consequences of maintained occlusion-induced vascular failure, both peripherally and centrally [19,23,24,27,29,31,37,38,39,40]. Consequently, as a chain of events that might be fully counteracted with BPC 157 therapy, there were counteracted in these rats with the occlusion and occlusion-like syndrome, the multiorgan failure (i.e., gastrointestinal, brain, heart failure, liver, and kidney lesions), portal and caval hypertension, aortal hypotension, intracranial (superior sagittal sinus) hypertension, and generalized thrombosis counteracted. This led to the useful BPC 157 therapy of the harmful circle, the counteraction of the generalized stasis, generalized Virchow triad presentation, and heart failure and severe ECG disturbances. As a prime and practical confirmation, rats with major vessel ligation and occlusion, in either artery and/or vein, and either peripherally or centrally, and other alike noxious occlusion-like procedures exhibited a similar syndrome (occlusion syndrome or occlusion-like syndrome) and shared full therapy benefit with the given BPC 157 therapy [18,19,23,24,27,29,31,37,38,39,40].

Evidently, BPC 157 vascular recovery therapy was able to provide adequate compensation (i.e., activation of collateral pathways to reestablish blood flow), both rapid and sustained, as demonstrated with BPC 157 therapy [18,19,23,24,27,29,31,37,38,39,40]. In support, there is an immense vascular network available for the rapid defense response that regularly failed, instead to be spontaneously activated. Thus, the BPC 157 therapy (i.e., endothelium function recovery and maintenance as innate cytoprotective effect) [8] might affect and reverse the shared innate inability to react spontaneously. Contrarily, not corrected, failed damaged endothelium function as an innate inability to react, would inevitably lead to the innate vascular and multiorgan failure and heart failure upon major vessel occlusion (ligation) as well as upon other similar noxious procedures (i.e., alcohol, lithium, isoprenaline, bile duct occlusion, maintained high intra-abdominal pressure) [18,19,23,24,27,29,31,37,38,39,40]. Given overwhelming thrombosis, peripherally and centrally, they might be seeable all as multiple occlusion syndrome, whether all vessels compressed (i.e., high intra-abdominal pressure [31]) or otherwise failed (i.e., occlusion [19,24,27,29], agent’s [38,39,40] or noxious other procedure [37] application), these might all be particular targets for BPC 157 bypassing key therapy.

## 3. Thrombosis

As mentioned before in the acute myocardial infarction and heart failure studies, counteraction of the harmful arrhythmias and thrombosis and the concomitant multiorgan failure and initiated multicausal noxious circuit that might also be counteracted all together might favor BPC 157 therapy as a particular cytoprotection application [18,19,22,23,24,25,27,28,29,30,31,37,38,39,40,41] (Table 2). The compelling evidence that against harmful thrombosis, BPC 157 might have a special beneficial effect [18,19,22,23,24,25,27,28,29,30,31,37,38,39,40,41] that might be utilized to reverse the heart failure cause–consequence, as occurred with the wide range of BPC 157 therapy, was specifically elaborated.

Commonly, illustrating thrombotic complications’ major role in patients with heart failure is a major issue in therapy; in 2021, several major trials attempted to resolve whether shortened dual antiplatelet therapy reduced bleeding risk without increasing the risk of further ischemic events [1].

On the other hand, it might be that BPC 157 (cytoprotection as endothelium function maintenance) [2,3,4,5,6,7,8] might collaborate with the evidence of heart failure innate etiopathology [163]. There are significant pro-thrombotic shifts and endothelial damage/dysfunction as a hallmark of heart failure, irrespective of any cause, related to the severity of heart failure [163]. Thereby, there are thrombi formations both within cardiac chambers (particularly in atrial fibrillation) and blood vessels, and both arterial endothelial dysfunction and venous dysfunction present in heart failure contribute to the pro-thrombotic state seen in this condition [163].

Given a special effect with BPC 157 therapy in the acute myocardial infarction and heart failure studies, the noted counteraction of the harmful arrhythmias and thrombosis and the concomitant multiorgan failure occurred simultaneously with the elimination/attenuation of the prominent hemorrhage and congestion in the many organs, such as brain, heart, lung, liver, kidney, and gastrointestinal tract [18,19,22,23,24,25,27,28,29,30,31,37,38,39,40,41].

Of note, conceptually, the cytoprotection rapidly went to the organoprotection (i.e., the stomach protection to the protection of other organs) [10,11,12,13,14,15,16,17], and in the particular BPC 157 case, the BPC 157 cytoprotection effect rapidly goes to the wound healing (i.e., implied direct cell protection against direct injury [10]) [3,8,102]. There is particular evidence noted with BPC 157 effects, in particular, wounding (i.e., abdominal aorta anastomosis [18] vs. amputation of the leg or tail [58,59,60], i.e., obstructing thrombus formation counteracted, and fully established the obstructing thrombus as rapidly annihilated [18] vs. decreased post-amputation bleeding [58,59,60]). Thus, we claimed that the realized healing effects in the various wound healing [53,55,103,104,105,106,107,108,109,110,111,112,113,114,115,116,117,118,119,120] might be evidence of the realized healing process after a ruptured blood vessel as a whole. Thereby, it might be the innate distinctive effect on all four major events in clot formation and dissolution fully accomplished that might be distinctively used depending on the given injury and agent application. This meant a special effect highly utilizable, possibly resolving the issue with heart failure therapy. Illustratively, BPC 157 therapy in rats with abdominal aorta anastomosis might prevent the occluding clot formation (early application soon after anastomosis creation) as well as annihilate already fully formed clot obstructing aorta (late application at 24 h after anastomosis creation). Simultaneously, BPC 157 therapy might both prevent leg disability and rapidly reestablish leg function [18]. Thus, there might be a well-functioning cytoprotection loop that might provide that the translation to the preserved muscle function consistently occurs.

BPC 157 attenuated the bleeding prolongation induced by anti-coagulants, anti-thrombotic agents, and NOS-substrate L-arginine, alone or with amputation (tail, leg), without affecting coagulation pathways [58,59,60]. Likewise, BPC 157 attenuated the bleeding from the leg or tail amputation [58,59,60], organ perforation or hemorrhagic mucosal lesions (cecum, stomach) [32,33], spinal cord compression [35,36], and intracerebral or intraventricular bleeding [19,24,27,29,31,37,38,39,40]. Furthermore, BPC 157 might specifically maintain the function of thrombocytes (as noted in aggregometry and thromboelastometry studies) [60]. Given with aspirin, clopidogrel, or cilostazol in rats, BPC 157 counteracted their inhibitory effects on aggregation activated by arachidonic acid, ADP, collagen, and arachidonic acid/PGE1 [60].

Providing strong interrelations between the arrhythmias, heart failure, and thrombosis [164], assuming the venous and arterial thrombosis as two aspects of the same disease [165,166], the BPC 157 counteracting effect might be reciprocally related. Thus, in heart failure therapy, the therapeutic effect of BPC 157 administration might counteract the escalating thrombosis as a shared common point [19,24,27,29,31,37,38,39,40]. This might be the consequent reversal of the general stasis (i.e., otherwise large volumes trapped in the damaged stomach, CNS, and portal and caval vein tributaries, which may also perpetuate the brain and heart ischemia). Along with this, it might be the reversal of the failed activation of the collateral bypassing pathways. This rapidly appeared within minutes while the major veins which had been disabled (inferior caval and superior mesenteric veins failed as congested and the azygos vein failed as collapsed [19,24,27,29,31,37,38,39,40]) might be quickly recovered, rapidly made fully functional. At the same time (i.e., direct blood delivery by the activated azygos vein), in the heart failure recovery, the otherwise progressing thrombosis in veins and arteries might be markedly attenuated (or even eliminated) as well as the progressing intracerebral and interventricular bleeding markedly attenuated or even annihilated [19,24,27,29,31,37,38,39,40]. Thus, this might be the innate resolution of the Virchow triad consequences [19,24,27,29,31,37,38,39,40]. Likely, this might be the effect related to the modulatory interaction with NO-system [61,62,63]. As proof, BPC 157 therapy effects might counteract in the same dosage range NOS-blockade (L-NAME)-induced pro-thrombotic and hypertensive effect as well as NOS-over-activity (L-arginine)-induced anti-thrombotic and hypotensive effect [59,62].

## 4. Blood Pressure

Interestingly, in patients who were hospitalized for heart failure, the risks of mortality and readmission increased at low and high blood pressures, with similar trends for patients with heart failure with reduced ejection fraction and with heart failure with preserved ejection fraction [167,168].

Severe blood pressure disturbances (i.e., intracranial (superior sagittal sinus), portal and caval hypertension, and aortal hypotension) were mentioned before to be attenuated/eliminated with BPC 157 therapy in the acute myocardial infarction, and in all heart failure studies [19,24,27,29,31,37,38,39,40]. These were along with noted counteraction of the harmful arrhythmias and thrombosis, and the concomitant multiorgan failure and initiated multicausal noxious circuit that might also be counteracted [19,24,27,29,31,37,38,39,40].

On the other hand, in the severe hyperkalemic condition regularly fatal within 30 min time, BPC 157 therapy, along with ascertained survival and counteraction of arrhythmias, might counteract hyperkalemia-induced hypertension [67]. Likewise, BPC 157 therapy might counteract hypertension induced by unilateral renal artery stenosis or by unilateral renal artery stenosis and contralateral nephrectomy [169]. Moreover, as mentioned before, BPC 157 therapy might counteract NOS-blocker L-NAME-induced hypertension, an effect along with counteraction of the L-NAME-induced pro-thrombotic effect [58,61,62]. Finally, in glaucomatous rats, BPC 157 might normalize the increased intraocular pressure [26].

Furthermore, BPC 157 therapy might oppose hypovolemic shock, hypotension, and mortality after controlled blood volume withdrawal [169]. Likewise, as mentioned before, BPC 157 therapy might counteract the NOS-substrate L-arginine-induced hypotension, an effect along with counteraction of the L-arginine-induced anti-thrombotic effect [58,61,62]. As in the mentioned acute studies, in the heart failure chronic studies with doxorubicin, along with counteraction of the heart failure, BPC 157 therapy strongly opposed hypotension [84]. This effect was along with the counteraction of the increased big endothelin-1 (BET-1) and plasma enzyme levels (CK, MBCK, LDH, AST, ALT) and improved clinical status in general [84].

Of note, low blood pressure is common in patients with heart failure and reduced ejection fraction [170]. The low blood pressure in heart failure with reduced ejection fraction shares multiple origins (i.e., low cardiac function, hypovolemia (i.e., diuretics (note, BPC 157 might counteract the harmful effects of furosemide overdose [69]), treatment-related vasodilatation, altered vasoreactivity (comorbidities, i.e., diabetes)) [171].

As a particular notation, BPC 157 had no effect on normal blood pressure [61,62]. Thus, the effect on blood pressure of BPC 157 therapy might be effectively related to the resolution of particular sick conditions, likely depending on the normalization of the heart function, as cytoprotection application is able to normalize either disturbed blood pressure or hypotension.

### Smooth Muscle

BPC 157 therapy might exert the described particular effect on blood pressure given the relaxation noted in the aorta without endothelium ex vivo but not relaxation directly on the 3D model composed of vascular smooth muscle cells (unlike the effect of NO-donor sodium nitroprusside) [53]. Possibly, this might be the release of the NO by its own [61,62,63], activated phosphorilazation of eNOS [53] as a special modulatory effect, given the mentioned counteraction of the adverse effect of NOS-blockade (i.e., L-NAME-hypertension and pro-thrombotic effect), as well as the counteraction of the adverse effect of NOS-over-stimulation (i.e., L-arginine-hypertension and anti-thrombotic effect) [58,62]. Moreover, the VEGFR2-Akt-eNOS signaling pathway might be activated without the need for other known ligands or shear stress, controlling vasomotor tone and the activation of the Src-Caveolin-1-eNOS pathway [53,54]. These might also be perceived as BPC 157/NO-system interaction in controlling blood pressure by a particular mechanism.

BPC 157 therapy might have a particular effect on other smooth muscles. During sick conditions, BPC 157 therapy might have a particularly beneficial effect on many sphincters (lower esophageal sphincter, pyloric sphincter [67,101,170,172,173,174,175,176,177,178], pupil [26,179], urinary sphincter [62,180,181]) and might recover their distinctive functions. This particular effect might suggest a distinctive therapy effect depending on the injury condition, along with the general agenda of the cytoprotection concept (i.e., maintained cell integrity against different noxious agents’ injurious effect) (for review, see, i.e., [2,3,4,5,6,7,8]). Moreover, it might maintain conditions of the sphincter’s normal functioning, modulating effect on distinctive sphincter functions, i.e., an anti-reflux effect (increases lower esophageal sphincter pressure, decreases pyloric sphincter pressure [170]) or maintained normal pupil diameter [179], or maintained normal leak point pressure [180].

## 5. Arrhythmias

In consideration of the BPC 157 therapy as a cytoprotection application, the counteraction of the arrhythmias was elaborated in the acute myocardial infarction and heart failure studies, along with the noted counteraction of the harmful thrombosis, and the concomitant multiorgan failure and initiated multicausal noxious circuit that might also be counteracted [19,24,27,29,31,37,38,39,40,41]. Further studies specifically address particular arrhythmias counteraction (Table 3). The BPC 157 therapy as antiarrhythmic agent follows the evidence that NO is commonly proposed as an endogenous cardioprotectant antifibrillatory factor [64,65] and that BPC 157 might modulate NO-effects (for review, see, i.e., [60]), and thereby might have a consistently strong beneficial effect against various arrhythmias and various agents and procedures that might produce arrhythmias [66,67,68,69,70,71,72]. Moreover, BPC 157 activities might approach and modulate the long-ago suggested antiarrhythmic agents potential throughout myocardial ischemia-arrhythmia-local anesthetic-anti-convulsion potential (for review, see, i.e., [182]).

### 5.1. Digitalis

Without therapy, the used digitalis regimen was regularly fatal, and thereby, it might be a particular challenge for the BPC 157 therapy, which might be dose-dependent, to both prevent or attenuate the development of the digitalis intoxication and reverse already established digitalis intoxication [66]. Without therapy, the established digitalis intoxication (the grade 3 AV-block quickly developed) outcome was inevitably complicated by fatal ventricular tachycardia and fatality in all animals.

In digitalis rats, AV-block might be a particular target for the BPC 157 therapy [66].

Given prophylactically, ng regimens reduced just the AV-block duration, while higher dose regimens, BPC 157 μg regimens, aside from AV-block, also reduced the number of ventricular premature beats, prolonged the time until the onset of ventricular tachycardia, and reduced the duration of ventricular tachycardia [66].

BPC 157 therapy completely changed the outcome of the established digitalis intoxication outcome. All BPC 157 regimens shortened the AV block and dose-dependently mitigated a further methyldigoxin-toxicity course. Ventricular tachycardias were avoided (50 μg/kg) or markedly reduced (10 μg/kg,10 ng/kg). Fatal outcomes were avoided (50 μg/kg), reduced (10 μg/kg), or only delayed (10 ng/kg). Most probably, these digitalis disturbances occurred as NO-related disturbances that might also be resolved with BPC 157 therapy [66].

Moreover, BPC 157 therapy also had the potential to compensate for the additional aggravation that might occur in the digitalis rats. Illustratively, the BPC 157 effect as NO-system related activity might evidence the BPC 157 administration to annihilate the strong aggravation that occurred with NOS-blocker L-NAME application, given either prophylactically or in the established digitalis intoxication [66].

### 5.2. Hyperkalemia

The hyperkalemia challenge for the BPC 157 therapy was intraperitoneal KCl-solution application (9 mEq/kg). In regularly deadly hyperkalemia (>12 mmol/L), arrhythmias with an ultimate and a regularly inevitable lethal outcome within 30 min [67] were both prevented with BPC 157 given before KCl application, as well as cured with BPC 157 therapy given later, in the conditions of the advanced and established huge hyperkalemia-induced disturbances. Intraperitoneal prophylactic regimen goes with the recovered sinus rhythm, less prolongation of QRS, and without asystolic pause [67]. Given at the 10 min point of the severely advanced downhill course after KCl application, the therapeutic regimen required 5–10 min period to start recovery, with normal sinus rhythm at 1 h. Of note, the particular BPC 157 counteracting potential toward hyperkalemia might first encourage the similar results obtained with the intragastric KCl application (27 mEq/kg)—(hyperkalemia 7 mmol/L) and BPC 157 therapy protocol (i.e., peaked T waves, fully counteracted by BPC 157 application, applied 30 min before or 10 min after KCl [67]). Then, the supporting point is the evidence that BPC 157 administration might have the potential to compete with further worsening instances. Illustratively, the direct effect seeable on potassium conductance in HEK293 cells, hyperkalemic conditions (18.6 mM potassium concentrations), counteracting the effect on membrane potential and depolarizations caused by hyperkalemic conditions, might annihilate L-NAME, NOS-blocker-induced aggravation, and thereby, BPC 157–hyperkalemia’s direct relationship might occur as a NO-system related interconnection. Likewise, other hyperkalemia disturbances (muscular weakness, hypertension, low sphincteric pressure with intraperitoneal KCl-application, severe stomach mucosal lesions, and sphincter failure with intragastric KCl-application) were also counteracted [67].

### 5.3. Succinylcholine

The counteraction/attenuation of the adverse effect of succinylcholine by BPC 157 therapy [68] might illustrate that, depending on the cause (potassium-overload [67]; succinylcholine application [68]), BPC 157 therapy (microgram and nanogram dose, intraperitoneal and peroral regimen) might distinctively affect hyperkalemia. While in the rats after potassium overload, the hyperkalemia persisted and the adverse effects were counteracted [67]. The illustrative might be the findings in the rats intramuscularly treated with succinylcholine (counteracted hyperkalemia, counteracted adverse effects). As succinylcholine acts as depolarizing neuromuscular blocker and disabling neuromuscular junction [68], this might indicate a particular recovering effect of BPC 157 therapy. Normokalemia and no arrhythmias, completely absent intermittent AV block and asystolic pauses, continuously maintained sinus rhythm, supplementing the evidence that BPC 157 therapy might attenuate the succinylcholine course as a whole. The therapeutic effect included the succinylcholine-induced behavioral agitation, muscle twitches, and motionless resting, and completely eliminated post-succinylcholine hyperalgesia, immediately eliminated leg contractures (intramuscular succinylcholine), counteracted both edema and the decrease in muscle fibers in the diaphragm and injected/non-injected anterior tibial muscles) [68]. Otherwise, succinylcholine-rats exhibited hyperkalemia with brisk arrhythmias (peaked T waves, widening of PR and QRS complexes, aggravation in intermittent AV block, and asystolic pauses (at 4–5 min period, but spontaneously recovered by the 15th min) [68].

### 5.4. Hypokalemia

The hypokalemia challenge for the BPC 157 therapy was a huge furosemide dose application and consequent efficacy in the otherwise deadly hypokalemia (<2.7 mmol/L) against the severe arrhythmias (i.e., polymorphic ventricular tachycardia (“torsades de pointes”)) (note, unlike full survival with BPC 157 therapy, without therapy, the lethal outcome occurred within 90–150 min) [69]. These were both prevented with BPC 157 given before furosemide application (AV block and abnormal ventricular rhythm were absent). Likewise, these were both annihilated with BPC 157 therapy given later after furosemide in the conditions of the advanced severe disturbances, i.e., the third-degree AV block and ventricular tachycardia, as complete restoration of the sinus rhythm occurred within a few minutes upon application of BPC 157 therapy [69]. In addition, the supporting point for BPC 157-hypokalemia particular relation is the evidence that BPC 157 administration might also counteract the further worsening. Illustratively, BPC 157 completely annihilated the aggravation induced by NOS-blocker L-NAME in the furosemide rats [69]. Moreover, BPC 157 therapy might have a direct effect on potassium conductance, seeable in HEK293 cells, BPC 157 (1 µM) abolished hyperpolarizations of HEK293 cells during hypokalemic (0.4 mM K) conditions [69]. Finally, as support of the effect on hypokalemia as a whole, all of the BPC 157 treated furosemide rats were without sudden, brief, shock-like, involuntary movements (i.e., hypokalemia-induced myoclonus), either completely prevented or rapidly reversed when they had been advanced along with arrhythmias [69].

Of note, the full practical significance of these BPC 157 findings [69] remained to be additionally elaborated (i.e., methyldigoxin toxicity depends particularly on hypokalemia [66]; variform ventricular tachycardia (“torsades de pointes“) also antagonized [69]). Furthermore, there is a strong working BPC 157 capability in either the hyperkalemic or hypokalemic conditions [67,68,69]. As such, these probably indicate the particular relations between the skeletal muscles (i.e., the largest single pool of K^+^ in the body [67,183]) and BPC 157 therapy (i.e., upon injury, strongly recovered skeletal muscle function and healing [110,111,112,113,114,115], recovered neuromuscular junction function [68]). These might have a considerable role in balancing the interconnected hyperkalemia/hypokalemia (i.e., hyperkalemia (i.e., exercise) is rapidly corrected by reaccumulation of potassium into the muscle cells via Na^+^, K^+^ pumps, often leading to hypokalemia [183]). Furthermore, BPC 157 might counteract the adverse effects (i.e., muscle weakness, brain lesions, myocardial infarction) of the overload with magnesium [184] or lithium [39] (both known to interfere with potassium functioning) [39,184]. Thus, BPC 157 might have a particularly beneficial effect as BPC 157 might also counteract the various forms of muscle weakness related to the large range of noxious events (i.e., those induced by tumor-cachexia [6], stroke [20], application of neurotoxins (cuprizone (mimicking multiple sclerosis) [185], 1-methyl-4-phenyl-1,2,3,6-tetrahydrophyridine (MPTP, mimicking Parkinson’s disease) [186]), or neuroleptics [187]). This might be decisive for the maintenance of muscle contractility and heart function.

### 5.5. Local Anesthetics, Bupivacaine

After an overdose of bupivacaine or any of the related amide local anesthetic agents, cardiovascular collapse, or even death, may occur [178]. Thereby, providing the used dose of bupivacaine (100 mg/kg IP), there is important evidence that BPC 157 successfully prevents and counteracts bupivacaine cardiotoxicity [70]. As a highlight of the practical applicability, BPC 157 is effective even against the worst outcomes, such as a severely prolonged QRS complex [70].

Amide local anesthetic agents overdose, in particular an overdose of bupivacaine (note, we used 100 mg/kg IP), might be associated with cardiovascular collapse or even death [188]. Likewise, BPC 157 therapy might be associated with counteraction of bupivacaine cardiotoxicity (i.e., bradycardia, high-degree AV-block, ventricular ectopies and tachycardia, T-wave elevation, respiratory arrest, and asystole), even with the upmost counteraction that might be needed against the worst outcomes (counteraction of the severely prolonged QRS complex). BPC 157 has no apparent limitation considering the therapy initiation. It might be effective early (at 30 min before or at 1 min after the bupivacaine injection, counteraction encompassed 50 μg/kg, 10 μg/kg, 10 ng/kg, or 10 pg/kg IP BPC 157 regimens). Likewise, it might be effective after delaying treatment (at 6 min after bupivacaine administration, and after the development of prolonged QRS intervals (20 ms), the fatal outcome was markedly postponed). Together, this might indicate a particular direct competition with the escalating bupivacaine course since, in HEK293 cells, BPC 157 inhibited the bupivacaine-induced depolarization [70]. Likewise, it remains to be seen how BPC 157 might specifically interfere with the specific bupivacaine inhibitory targets, such as the transient outward K+ current in ventricular myocytes or the fast block of sodium channels during the action potential with slow recovery from block during diastole [189,190].

### 5.6. Local Anesthetics, Lidocaine

The antagonism of the entire spectrum of local anesthetic-induced neurotoxic and cardiotoxic effects [71] was the issue with lidocaine, as prototype application (intraperitoneal, intraplantar and axillary, and spinal (L4-L5) intrathecal block) toward the particular beneficial recovering effect of BPC 157 therapy, intraplantar, intraperitoneal, and intragastric application. First, BPC 157 counteracted lidocaine–bradycardia, which might be a severe one, prevented bradycardia development as well as reversed established bradycardia, given either before or after lidocaine [71]. Likewise, BPC 157, given before or after, counteracted the lidocaine-induced local anesthesia via the intraplantar application and axillary and spinal (L4-L5) intrathecal block. Moreover, BPC 157 counteracted lidocaine-induced convulsions. In vitro, BPC 157 counteracted lidocaine-induced HEK293 cell depolarization [71]. There might be BPC 157-lidocaine-NO-system interconnections [71], providing that BPC 157 administration might completely annihilate the strong aggravation that might occur with the NOS-blocker L-NAME application [71].

### 5.7. Neuroleptics and Prokinetics Induced Prolonged QTc Interval

The evidence of the beneficial BPC 157 effect with the neuroleptics and prokinetics application versus the prolonged QTc intervals is a known shared adverse effect of the neuroleptics and prokinetics application [72]. Here, with the prolonged QTc intervals effect after application of the dopamine neuroleptics and prokinetic metoclopramide, but not after domperidone (known to act peripherally), the neuroleptics and prokinetic prolonged QTc intervals occurred as the particular central effect [72]. Finally, the consistent antagonization and the use of the various neuroleptics, both typical (haloperidol, fluphenazine) and atypical (sulpiride, clozapine, quetiapine), might provide the BPC 157 therapy potential (i.e., the counteraction of the prolonged QTc intervals) as capable of antagonizing an essential class adverse effect [72]. Note that the potential involvement of pathological ion channel modulation might be shared disorder in the etiology of neurological disorders, cardiovascular disease, and, ultimately, arrhythmias [191]. Likewise, in the same dose regimens, BPC 157 therapy counteracted the neuroleptic-induced catalepsy and akinesia and gastrointestinal disturbances [173,192] and hippocampal ischemia/reperfusion injuries in rats in the stroke studies (therapy after reperfusion initiation, after carotid arteries clamping) [20]. Additionally, with BPC 157 therapy, there might be the brain–gut and gut–brain axis function recovery [2,193], while BPC 157, when given peripherally, might exert particular central beneficial effects [2,193]. Illustratively, these were the release of serotonin in the specific brain areas (i.e., nigrostriatum) [194], as opposed to the schizophrenia-like positive symptoms models [187] and schizophrenia-like negative symptoms models [195]. Finally, BPC 157 counteracted various encephalopathies [91,92,93,94,95,96].

Thus, as a particular point of the BPC 157 activities (i.e., BPC 157 counteracted lidocaine arrhythmias, local anesthetic effect, and convulsions [72]), it might be that BPC 157 activities might approach and modulate the long-ago suggested antiarrhythmic agents potential throughout myocardial ischemia-arrhythmia-local anesthetic-anti-convulsion potential (for review, see, i.e., [182]). Illustratively, in addition to the lidocaine-induced convulsion antagonization, BPC 157 might counteract standard convulsants (i.e., picrotoxine, strychnine, bicuculline, metrazole)-induced seizures [169], as well as the insulin-[96], paracetamol [91], alcohol withdrawal [196] and serotonin syndrome-[197] induced convulsion. In addition to the lidocaine-local anesthetic effect [71], BPC 157 also counteracted tetracaine and the oxybuprocaine effect on corneal anesthesia [198] and bupivacaine severe arrhythmias [70]. BPC 157 has an analgesic effect of its own [199,200,201,202]. Illustratively, BPC 157 produced analgesia in the MgSO_4_ and acetic acid test in mice, a model of prolonged pain associated with tissue injury [199], counteracted succinylcholine muscle pain (violent screaming upon light touch) in rats [68], and intra-articular injection of BPC 157 for multiple types of knee pain in patients [201], likely as a part of its particular healing effect). Namely, on the other hand, it might antagonize the morphine-analgesia and haloperidol potentiation of the morphine-analgesia [203].

In summary, the arrhythmias/BPC 157 evidenced direct effect demonstrated in vivo and in vitro studies might reveal a quite large range of the arrhythmias [66,67,68,69,70,71,72,73] that BPC 157 might counteract. The counteracting therapy effect occurred throughout even opposite circumstances (i.e., hyperkalemia vs. hypokalemia; hyperkalemia-depolarization vs. hypokalemia-hyperpolarization) and throughout particular targets (i.e., Na^+^, K^+^ pump (digitalis), sodium channels (local anesthetics), potassium channels (hypokalemia/hyperkalemia, succinylcholine), dopamine receptors (neuroleptics)) [66,67,68,69,70,71,72,73]. With BPC 157 therapy, all of the heart arrhythmias might be equally affected, as might also be affected the other noxious effects that occurred along with arrhythmias [19,24,27,29,31,37,38,39,40,41,66,67,68,69,70,71,72,73]. BPC 157 therapy intercepting cardiac arrhythmias should be quite extensive, especially considering the wide range of the additional adverse effects [19,24,27,29,31,37,38,39,40,41,66,67,68,69,70,71,72,73] that were also counteracted. This might be understood through a particular modulatory effect, direct cytoprotective cell protection (reestablished cell membrane potential in vitro, counteracted both hyperkalemia-depolarization and hypokalemia-hyperpolarization) [19,24,27,29,31,37,38,39,40,41,66,67,68,69,70,71,72,73], that might, in general, compensate and maintain (cardiac) cell integrity and function against the failed circumstances created by the persisting noxious event (i.e., hyperkalemia, hypokalemia, different noxious agents application) [19,24,27,29,31,37,38,39,40,41,66,67,68,69,70,71,72,73]. In general, the entire myocardial cell cytoprotective effect might mean the entire antiarrhythmic effect. In addition to the mentioned BPC 157/NO-system relation [61], this particular modulating cytoprotective point might be an innate defensive response that might resolve the still-existing paradox that antiarrhythmic drugs can also generate arrhythmias.

In general, all antiarrhythmic drugs in current use have various shortcomings, and neither is free from considerable side effects of one kind or another. Thereby, summarizing the findings of the antidysrhythmic effects of the BPC 157 therapy application, a particularly beneficial effect might be envisaged. It seemed that it might beneficially affect all groups of arrhythmias (i.e., extra beats, supraventricular tachycardias, ventricular arrhythmias, and bradyarrhythmias). Moreover, in a more general view, decisive for the maintenance of muscle contractility and heart function, the counteraction of arrhythmias as an interaction of several changes in the fundamental electrophysiological properties of cardiac muscle fibers might also consider the additional BPC 157 therapy effect. These might be the simultaneous beneficial recovery of the disabled striated muscle function [67,68,69,71,72,111,112,113,114,115] as well as recovery of the disabled smooth muscle [101,170,172,173,174,175,176,177,178,179,180,181], effectively working through hyperkalemic [67,68] and hypokalemic [69] conditions. Evidently, BPC 157 therapy annihilated lethal outcomes in both hyperkalemia [67] and hypokalemia [69]). Finally, for the large antidysrhythmic effects of the BPC 157 therapy [19,22,23,24,25,27,28,29,30,31,37,38,39,40,41,66,67,68,69,70,71,72,73], the counteraction of the local myocardial ischemia consistently encountered with BPC 157 therapy as part of its cytoprotective effect [18,19,22,23,24,25,27,28,29,30,31,37,38,39,40,41] might be responsible.

## 6. Conclusions

**Figure 1:**
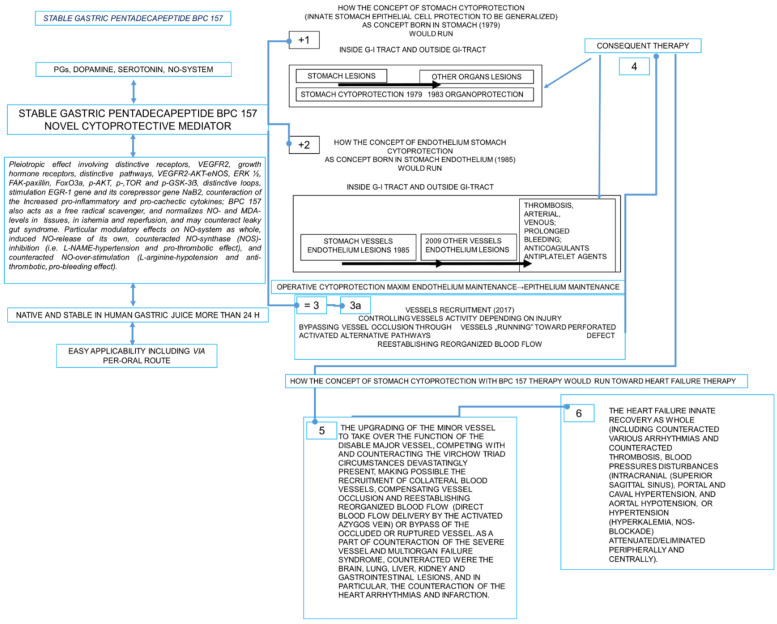
Description of the BPC 157 therapy. Cytoprotection concept (for review, see [2,3,4,5,6,7,8,10,11,12,13,14,15,16,17]) born in the stomach appears with cytoprotective agents application as innate stomach epithelial cell protection (indicated as +1) to be generalized (Robert, Szabo) in other organs epithelia protection (organoprotection) supplemented by stomach endothelium cell protection (indicated as +2) (Szabo). Together (+1, +2), these result in cytoprotection stomach maxim endothelial maintenance→epithelial maintenance (indicated as = 3) as axis for the rapid defensive response to resolve the ongoing lesions, which is, however, not fully operative with the standard cytoprotective agents. As novel cytoprotection mediator, BPC 157 might exert prominent epithelial beneficial effects in the stomach and in the whole gastrointestinal tract and in the other organs (stomach cytoprotection→organoprotection) (+1) and endothelial beneficial effect in the stomach (+2). Therefore, BPC 157 therapy might make the stomach maxim endothelial maintenance→epithelial maintenance (indicated as =3) a fully operative axis (indicated as 3a). Further, BPC 157 therapy might extend the original cytoprotection maxim endothelial maintenance→epithelial maintenance from the stomach to the other vessels endothelium protection (3a) [18,19,24,27,29,31,37,38,39,40,41]. In this, BPC 157 might induce particular vessel recruitment and activation depending on injury, i.e., when confronted with vessel occlusion, there was collateral activation to bypass vessel occlusion, as well as when confronted with perforated defect, vessel “running“ toward the defect (3a) [18,19,24,27,29,31,37,38,39,40,41]. The rapid result is the re-establishing of the reorganized blood flow (3a). As consequence, there was a particular therapy that might beneficially affect thrombosis, arterial and venous, and lesions presentation (indicated as 4). For the BPC 157 therapy of the heart failure, BPC 157 therapy might induce particular upgrading of the minor vessel to take over function of the disabled major vessel, resolving Virchow triad circumstances devastatingly present, making possible collateral vessels activation, compensating function of the major vessel, reestablishing reorganized blood flow (direct blood flow by the activated azygos vein) (indicated as 5) [18,19,24,27,29,31,37,38,39,40,41]. In confrontation with the severe syndrome (i.e., heart failure, in particular), the successful activation of the compensatory collateral circulation was ascribed to the counteraction of the vascular and multiorgan failure, counteraction of the intracranial (superior sagittal sinus), portal and caval hypertension and aortal hypotension (5) [18,19,24,27,29,31,37,38,39,40,41]. The final result of the BPC 157 therapy (indicated as 6) might be the heart failure innate recovery as a whole (including counteracted various arrhythmias and counteracted thrombosis, blood pressures disturbances (intracranial (superior sagittal sinus), portal and caval hypertension, and aortal hypotension [19,24,27,29,31,37,38,39,40,84], or hypertension (hyperkalemia, NOS-blockade) [62,67] attenuated/eliminated peripherally and centrally). Moreover, as part of the general beneficial pleiotropic effect (as part of the cytoprotection background) [2,3,4,5,6,7,8], there was the counteraction of the concomitant severe vessel and multiorgan failure syndrome [19,24,27,29,31,37,38,39,40], the counteraction of the brain, lung, liver, kidney and gastrointestinal severe lesions, almost annihilated thrombosis, counteraction of the escalated general peripheral and central syndrome. In addition, BPC 157 also acts as a free radical scavenger, counteracts free radical-induced lesions, and normalizes NO and MDA levels in tissues and during ischemia and reperfusion [5,6,19,22,24,25,27,28,29,30,32,33,34,38,39,90,160,161], and thereby, due to its particular cytoprotective/cardioprotective activity [2,3,4,5,6,7,8], it might beneficially affect the myocardial lesions, in particular [19,24,27,29,31,37,38,39,40,41,84]. Finally, in addition to the clear demonstration of the therapeutic efficacy in the most adequate animal models as proof of the concept, the additional evidence involves particular interaction with many molecular pathways [2,3,4,5,6,7,8,20,22,50,51,52,53,54,55,56,57]. Pleiotropic effects involving distinctive receptors, including VEGFR2 and growth hormone receptors, distinctive pathways, including VEGFR2-AKT-eNOS, ERK ½, FAK-paxillin, FoxO3a, p-AKT, p-mTOR and p-GSK-3β, and distinctive loops, including stimulation of the egr-1 gene and its corepressor gene naB2, and counteraction of increases in pro-inflammatory and procachectic cytokines, and counteraction of the leaky gut syndrome [2,3,4,5,6,7,8,20,22,50,51,52,53,54,55,56,57], likely minimize the inherent lack of full understanding of the mechanisms that may be involved. In the interaction with many molecular pathways [2,3,4,5,6,7,8,20,22,50,51,52,53,54,55,56,57], particular consideration should be given to the evidence of the BPC 157/ NO-system’s particular importance (i.e., the endothelium and thrombocytes function both maintained (for review, see, i.e., [2,3,4,5,6,7,8])). BPC 157 therapy counteracted thrombocytopenia in rats that underwent major vessel occlusion and deep vein thrombosis [22] and counteracted thrombosis in all vascular studies [18,19,23,24,27,28,29,31,37,38,39,40]), and coagulation pathways not affected [58,59,60]. Further arguments might be controlling vasomotor tone and the activation of the Src-Caveolin-1-eNOS pathway [53,54]. This likely occurred as the particular modulatory effects on NO-system as a whole, induced NO-release of its own [61,62,63], counteracted NOS-inhibition [61] (i.e., N(G)-nitro-L-arginine methylester (L-NAME)-hypertension and pro-thrombotic effect) [58,62], and counteracted NO-over-stimulation [61] (L-arginine-hypotension and anti-thrombotic, pro-bleeding effect) [58,62]. Likewise, the isoprenaline-myocardial infarction was counteracted as NO-effect [38]. Thus, due to its close interaction with NO-system as NO acts as an endogenous cardioprotectant antifibrillatory factor [64,65] and BPC 157 might have a particular therapeutic effect.

As the essential new result of the stable gastric pentadecapeptide BPC 157 therapy, as a part of the implemented cytoprotective effect (see Section 1, Section 1.1 and Section 1.2), specifically proved in the vascular studies, there was the particular activation of the collateral pathways [18,19,20,21,22,23,24,25,26,27,28,29,30,31,32,33,34,35,36,37,38,39,40,41]. Illustratively, in the rats with myocardial infarction and heart failure, the azygos vein might be completely collapsed (failed collateral pathways), while with BPC 157 therapy activated the azygos vein which might provide direct blood flow delivery to the superior caval vein and reestablish reorganized blood flow [19,24,27,29,31,37,38,39,40]. With BPC 157 therapy, this might be the upgrading of the minor vessel to take over the function of the disabled major vessel, competing with and resolving the Virchow triad circumstances, which might be devastatingly present (i.e., almost annihilated thrombosis as a positive outcome of the regained endothelium function and resolved stasis). This might make possible the recruitment of collateral blood vessels, compensating vessel occlusion, and reestablishing blood flow or bypassing the occluded or ruptured vessel [18,19,20,21,22,23,24,25,26,27,28,29,30,31,32,33,34,35,36,37,38,39,40,41]. Thereby, the described BPC 157 therapy beneficial effects (see Section 2, Section 2.1, Section 2.2, Section 3, Section 4 and Section 5) might be seen as a network of the interrelated evidence that together might support each other effect for the physiologic significance of the revealed BPC 157/vascular-system interplay. As a part of the effectively realized BPC 157/vascular-system interplay (lack of the adverse effect), there was the heart failure innate recovery as a whole (including counteracted various arrhythmias and counteracted thrombosis, blood pressures disturbances (intracranial (superior sagittal sinus), portal and caval hypertension, and aortal hypotension [19,24,27,29,31,37,38,39,40,84], or hypertension (hyperkalemia, NOS-blockade) [62,67] attenuated/eliminated peripherally and centrally). Moreover, as part of the general beneficial pleiotropic effect (as part of the cytoprotection background) [2,3,4,5,6,7,8], there was the counteraction of the concomitant severe vessel and multiorgan failure syndrome [19,24,27,29,31,37,38,39,40], the counteraction of the brain, lung, liver, kidney and gastrointestinal severe lesions, almost annihilated thrombosis, counteraction of the escalated general peripheral and central syndrome. Thus, given the obtained beneficial effects in the heart failure of the BPC 157 therapy and the counteraction of the concomitant pathology, it might be that the heart failure cause–consequence circuit might occur in a multidirectional way that BPC 157 therapy might beneficially affect as a whole. Centrally, illustrative heart failure cause–consequence circuit [27] goes as the comparable BPC 157 therapy effect on the stroke in rats, therapy in the reperfusion after bilateral clamping of the common carotid arteries for a 20-min period [20]. At the periphery, the heart failure cause–consequence circuit might occur between the heart and affected organ (i.e., lung, liver, kidney, gastrointestinal tract) in a multidirectional way that might be all beneficially affected by the BPC 157 therapy [19,24,27,29,31,37,38,39,40]. Finally, the physiologic significance of the revealed BPC 157/vascular-system interplay goes with the BPC 157 found in situ hybridization and immunostaining studies in humans to be largely distributed in tissues [3,102], and similar effects and roles in other species (i.e., birds [86], and insects, honeybees [204,205]). There might also be additional physiologic regulatory roles [3,102] (i.e., plethora interactions with distinctive molecular pathways [50,51,52,53,54,55,56,57] (in particular NO-system [53,54,61,62,63] and prostaglandin-system [2,3,4,5,6,7,8,74]), throughout the healing and vascular recovery). These might be along with the very safe BPC 157 profile (i.e., absent adverse effects in clinical trials (ulcerative colitis, phase II), LD1 could be not achieved in toxicological studies) (for review, see, i.e., [2,3,4,5,6,7,8,61,74,102]). This might be taken as a definitive advantage, as recently confirmed in a large study conducted by Xu and collaborators [206]. Together, these findings (for review, see, i.e., [2,3,4,5,6,7,8,61,74,102]) are suggestive of the BPC 157 cytoprotection application in further vascular injuries therapy, and suitable for use on myocardial infarction, heart failure, pulmonary hypertension, arrhythmias, and thrombosis therapy as well (for a suited summary see concluding Figure 1).

Stable gastric pentadecapeptide BPC 157 is a partial sequence of the human gastric juice protein BPC, which is freely soluble in water at pH 7.0 and in saline. BPC 157 (GEPPPGKPADDAGLV, molecular weight 1419; Diagen, Slovenia) was prepared as a peptide with 99% high-performance liquid chromatography (HPLC) purity, with 1-des-Gly peptide being the main impurity. PGs, prostaglandins; NO, nitric oxide; VEGF, vascular endothelial growth factor; VEGFR2, VEGF receptor 2; eNOS, endothelial nitric oxide synthase; FAK, focal adhesion kinase; FoxO3a, transcription factor; p-AKT, phospho-AKT; p-mTOR, phospho mammalian target of rapamycin; p-GSK-3β, phospho glycogen synthase kinase 3β; MDA, malondialdehyde; GI, gastrointestinal.

## Figures and Tables

**Table 1:** Summarized presentation of the BPC 157 therapy effect on myocardial infarction and heart failure that were induced in the vascular studies [19,24,27,29,31,37,38,39,40,41].

Noxious Procedure	BPC 157 Therapy Effects
Initial heart infarct induction and re-infarction, isoprenaline one or two application Escalated general peripheral and central syndrome [38]	Reduced levels of all necrosis markers, CK, CK-MB, LDH, and cTnT, and attenuated gross (no visible infarcted area) and histological damage, ECG (no ST-T ischemic changes), and echocardiography (preservation of systolic left ventricular function) damage induced by isoprenaline. Decrease in oxidative stress parameters and likely maintained NO-system function evidenced that BPC 157 interacted with eNOS and COX2 gene expression in a particular way and counteracted the noxious effect of the NOS-blocker, L-NAME. Early vessel and multiorgan failure (brain, heart, lung, liver, kidney, and gastrointestinal lesions), thrombosis, intracranial (superior sagittal sinus) hypertension, portal and caval hypertension, and aortal hypotension, ECG disturbances), in its full presentation was attenuated/eliminated by BPC 157 therapy (given at 5 min after isoprenaline) via activation of the azygos vein).
Intragastric administration of 96% alcohol Escalated general peripheral and central syndrome acute subendocardial infarct [40]	Intragastric administration of absolute alcohol-induced gastric lesions, intracranial (superior sagittal sinus) hypertension, severe brain swelling and lesions (i.e., intracerebral hemorrhage with degenerative changes of cerebral and cerebellar neurons), portal and vena caval hypertension, aortal hypotension, severe thrombosis, inferior vena cava and superior mesenteric vein congestion, azygos vein failure (as a failed collateral pathway), electrocardiogram disturbances, and heart (acute subendocardial infarct), lung (parenchymal hemorrhage), liver (congestion), and kidney (congestion) lesions. BPC 157 therapy (10 µg/kg or 10 ng/kg given intraperitoneally 1 min after alcohol) counteracted these deficits rapidly. Specifically, BPC 157 reversed brain swelling and superior mesenteric vein and inferior vena caval congestion and helped the azygos vein to recover, which improved the collateral blood flow pathway.
Lithium sulfate regimen in rats (500 mg/kg/day, ip, for three days, with assessment at 210 min after each administration of lithium) Escalated general peripheral and central syndrome Severe myocardial congestion, along with subendocardial infarcts [39]	BPC 157 counteracted the lithium-induced occlusive-like syndrome; rapidly counteracted brain swelling and intracranial (superior sagittal sinus) hypertension, portal hypertension, and aortal hypotension, which otherwise would persist; counteracted vessel failure; abrogated congestion of the inferior caval and superior mesenteric veins; reversed azygos vein failure; and mitigated thrombosis (superior mesenteric vein and artery), congestion of the stomach, and major hemorrhagic lesions. Both regimens of BPC 157 administration also counteracted the muscular weakness and prostration (as shown in microscopic and ECG recordings), myocardial congestion and infarction, in addition to edema and lesions in various brain areas; counteracted marked dilatation and central venous congestion in the liver; large areas of congestion and hemorrhage in the lung; and degeneration of proximal and distal tubules with cytoplasmic vacuolization in the kidney, attenuating oxidative stress.
Abdominal compartment syndrome (intra-abdominal pressure in thiopental-anesthetized rats at 25 mmHg (60 min), 30 mmHg (30 min), 40 mmHg (30 min), and 50 mmHg (15 min), and, in esketamine-anesthetized rats (25 mmHg for 120 min)) as a model of multiple occlusion syndrome Escalated general peripheral and central syndrome Severe myocardial congestion, along with subendocardial infarcts [31]	BPC 157 administration recovered the azygos vein via the inferiorsuperior caval vein rescue pathway. Additionally, intracranial (superior sagittal sinus), portal, and caval hypertension and aortal hypotension were reduced, as were the grossly congested stomach and major hemorrhagic lesions, brain swelling, venous and arterial thrombosis, congested inferior caval and superior mesenteric veins, and collapsed azygos vein; thus, the failed collateral pathway was fully recovered. Severe ECG disturbances (i.e., severe bradycardia and ST-elevation until asystole) were also reversed. Microscopically, transmural hyperemia of the gastrointestinal tract, intestinal mucosa villi reduction, crypt reduction with focal denudation of superficial epithelia, and large bowel dilatation were all inhibited. In the liver, BPC 157 reduced congestion and severe sinusoid enlargement. In the lung, a normal presentation was observed, with no alveolar membrane focal thickening and no lung congestion or edema, and severe intra-alveolar hemorrhage was absent. Moreover, severe heart congestion, subendocardial infarction, renal hemorrhage, brain edema, hemorrhage, and neural damage were prevented.
Bile duct ligation acute pancreatitis as local disturbances (i.e., improved gross and microscopy presentation, decreased amylase level) Escalated general peripheral and central syndrome Severe myocardial congestion, along with subendocardial infarcts [37]	Bile duct-ligated rats commonly presented intracranial (superior sagittal sinus), portal and caval hypertension and aortal hypotension, gross brain swelling, hemorrhage and lesions, heart dysfunction, lung lesions, liver and kidney failure, gastrointestinal lesions, and severe arterial and venous thrombosis, peripherally and centrally. Unless antagonized with the key effect of BPC 157 regimens, reversal of the inferior caval and superior mesenteric vein congestion and reversal of the failed azygos vein activated azygos vein-recruited direct delivery to rescue the inferior-superior caval vein pathway; these were all antecedent to acute pancreatitis major lesions (i.e., acinar, fat necrosis, hemorrhage). These lesions appeared in the later period but were markedly attenuated/eliminated (i.e., hemorrhage) in BPC 157-treated rats. To summarize, while the innate vicious cycle may be peripheral (bile duct ligation), or central (rapidly developed brain disturbances), or peripheral and central, BPC 157 resolved acute pancreatitis and its adjacent syndrome.
Superior mesenteric artery permanent occlusion Escalated general peripheral and central syndrome Severe myocardial congestion [19]	BPC 157 rapidly recruits collateral vessels (inferior anterior pancreaticoduodenal artery and inferior mesenteric artery) that circumvent occlusion and ascertains blood flow distant from the occlusion in the superior mesenteric artery. Portal and caval hypertension, aortal hypotension, and, centrally, superior sagittal sinus hypertension were attenuated or eliminated, and ECG disturbances were markedly mitigated. BPC 157 therapy almost annihilated venous and arterial thrombosis. Multiple organ lesions and disturbances (i.e., heart, lung, liver, and gastrointestinal tract, in particular, as well as brain) were largely attenuated.
Irremovable occlusion of the end of the superior mesenteric vein Escalated general peripheral and central syndrome Severe myocardial congestion [24]	BPC 157 rapidly activated the superior mesenteric vein-inferior anterior pancreaticoduodenal vein-superior anterior pancreaticoduodenal vein-pyloric vein-portal vein pathway, reestablished superior mesenteric vein and portal vein connection and reestablished blood flow. Simultaneously, toward inferior caval vein, an additional pathway appears via the inferior mesenteric vein, united with the middle colic vein, throughout its left colic branch to ascertain alternative bypassing blood flow. Consequently, BPC 157 acts peripherally and centrally and counteracts the intracranial (superior sagittal sinus), portal and caval hypertension, aortal hypotension, ECG disturbances attenuated, abolished progressing venous and arterial thrombosis. Additionally, BPC 157 counteracted multiorgan dysfunction syndrome, heart (severe myocardial congestion), lung, liver, kidney, gastrointestinal tract, brain lesions, and oxidative stress in tissues.
Permanently occluded essential vessel tributaries, both arterial and venous, occluded superior mesenteric vein and artery in rats Escalated general peripheral and central syndrome Severe myocardial congestion, along with subendocardial infarcts [29]	BPC 157 rapidly activated collateral pathways. These collateral loops were the superior mesenteric vein-inferior anterior pancreaticoduodenal vein-superior anterior pancreaticoduodenal vein-pyloric vein-portal vein pathway, an alternative pathway toward inferior caval vein via the united middle colic vein and inferior mesenteric vein through the left colic vein, and the inferior anterior pancreaticoduodenal artery and inferior mesenteric artery. Consequently, BPC 157 counteracted the superior sagittal sinus, portal and caval hypertension, aortal hypotension, progressing venous and arterial thrombosis peripherally and centrally, ECG disturbances attenuated. Markedly, the multiple organ lesions, heart, lung, liver, kidney, and gastrointestinal tract, in particular, as well as brain lesions and oxidative stress in tissues, were attenuated.
Complex syndrome of the occluded superior sagittal sinus, brain swelling and lesions, and multiple peripheral organs lesions in rat Escalated general peripheral and central syndrome Severe myocardial congestion [27]	The increased pressure in the superior sagittal sinus, portal and caval hypertension, aortal hypotension, arterial and venous thrombosis, severe brain swelling and lesions (cortex (cerebral, cerebellar), hypothalamus/thalamus, hippocampus), particular veins (azygos, superior mesenteric, inferior caval) dysfunction, heart dysfunction, lung congestion as acute respiratory distress syndrome, kidney disturbances, liver failure, and hemorrhagic lesions in gastrointestinal tract were all assessed. Rats received BPC 157 medication (10 µg/kg, 10 ng/kg) intraperitoneally, intragastrically, or topically to the swollen brain at 1 min ligation time or at 15 min, 24 h, and 48 h ligation time. BPC 157 therapy rapidly attenuates the brain swelling, rapidly eliminates the increased pressure in the ligated superior sagittal sinus and the severe portal and caval hypertension and aortal hypotension, and rapidly recruits collateral vessels, centrally ((para)sagittal venous collateral circulation) and peripherally (left superior caval vein azygos vein-inferior caval vein). BPC 157 therapy rapidly overwhelms the permanent occlusion of the superior sagittal sinus in rats and counteracts the brain, heart, lung, liver, kidney, and gastrointestinal lesions, and annihilates thrombosis, given at 1 min, 15 min, 24 h, or 48 h ligation-time.
Monocrotaline-induced pulmonary arterial hypertension in rats (wall thickness, total vessel area, heart frequency, QRS axis deviation, QT interval prolongation, increase in right ventricle systolic pressure, and body weight loss) [41]	After monocrotaline (80 mg/kg subcutaneously), BPC 157 (10 μg/kg or 10 ng/kg, days 1–14 or days 1–30 (early regimens), or days 14–30 (delayed regimen)) was given once daily intraperitoneally (last application 24 h before sacrifice) or continuously in drinking water until sacrifice (day 14 or 30). Without therapy, the outcome was the full monocrotaline syndrome, marked by right-side heart hypertrophy and massive thickening of the precapillary artery’s smooth muscle layer, clinical deterioration, and sometimes death due to pulmonary hypertension and right-heart failure during the fourth week after monocrotaline injection. With all BPC 157 regimens, monocrotaline-induced pulmonary arterial hypertension (including all disturbed parameters) was counteracted, and consistent beneficial effects were documented during the whole course of the disease. Pulmonary hypertension was not even developed (early regimens) as quickly as advanced pulmonary hypertension was rapidly attenuated and then completely eliminated (delayed regimen).
Congestive heart failure after doxorubicin regimen (total dose of 15 mg/kg intraperitoneally, divided at six time points, every third day for 14 days to induce congestive heart failure). After four weeks of rest, assessed in mice and rats with advanced disease course, the increased big endothelin-1 (BET-1) and plasma enzyme levels (CK, MBCK, LDH, AST, ALT), before and after next subsequent fourteen days of therapy, and clinical status (hypotension, increased heart rate and respiratory rate, and ascites) every two days [84].	Without therapy, throughout 14 days, both rats and mice further raised BET-1 serum values and aggravated clinical status, while enzyme values maintained their initial increase. BPC 157 (10 µg/kg) and amlodipine treatment reversed the increased BET-1 (rats, mice), AST, ALT, CK (rats, mice), and LDH (mice) values. BPC 157 (10 ng/kg) and losartan opposed further increase of BET-1 (rats, mice). Losartan reduces AST, ALT, CK, and LDH serum values. BPC 157 (10 ng/kg) reduces AST and ALT serum values. Clinical status of chronic heart failure in rats and in mice is accordingly improved by the BPC 157 regimens and amlodipine. However, indicatively, translation to the counteracted hypotension, no dyspnea with increased heart and respiratory occurred in BPC 157 treated animals, whereas hypotension and dyspnea with increased heart rate and respiratory rate persisted in the losartan and amlodipine treated animals.

**Table 2:** BPC 157 administration might counteract the escalating thrombosis as a particular commonly shared point.

Applied Noxious Procedure and BPC 157 Therapy Effect	
Attenuated/Eliminated Arterial and Venous Thrombosis	Attenuated/Eliminated Bleeding
Abdominal aorta anastomosis in rats. *J. Physiol. Pharmacol.* **2009**, *60 Suppl 7*, 161–165. [18]	Infrarenal inferior caval vein occlusion in rats. *Vascul. Pharmacol.* **2018**, *106*, 54–66. [18]
Infrarenal inferior caval vein occlusion in rats. *Vascul. Pharmacol.* **2018**, *106*, 54–66. [22]	Suprahepatic occlusion of the inferior caval vein, Budd-Chiari syndrome model in rats. *World J. Gastrointest. Pathophysiol.* **2020**, *11*, 1–19. [23]
Suprahepatic occlusion of the inferior caval vein, Budd-Chiari syndrome model in rats. *World J. Gastrointest. Pathophysiol.* **2020**, *11*, 1–19. [23]	Pringle maneuver in rats, both ischemia and reperfusion. *World J. Hepatol.* **2020**, *12*, 184–206. [28]
Pringle maneuver in rats, both ischemia and reperfusion. *World J. Hepatol.* **2020**, *12*, 184–206. [28]	Occlusion of the superior mesenteric artery in rats. *Biomedicines* **2021**, *9*, 609. [19]
Occlusion of the superior mesenteric artery in rats. *Biomedicines* **2021**, *9*, 609. [19]	Occlusion of the end of the superior mesenteric vein in rats. *Biomedicines* **2021**, *9*, 1029. [24]
Occlusion of the end of the superior mesenteric vein in rats. *Biomedicines* **2021**, *9*, 1029. [24]	Occluded superior mesenteric artery and vein in rats. *Biomedicines* **2021**, *9*, 792. [29]
Occluded superior mesenteric artery and vein in rats. *Biomedicines* **2021**, *9*, 792. [29]	Occlusion of the superior sagittal sinus in rats. *Biomedicines* **2021**, *9*, 744. [27]
Occlusion of the superior sagittal sinus in rats. *Biomedicines* **2021**, *9*, 744. [27]	Perforated cecum lesions in rats. *World J. Gastroenterol.* **2018**, *24*, 5462–5476. [32]
Acute pancreatitis as vascular failure-induced severe peripheral and central syndrome in rats. *Biomedicines.* **2022**, *10*, 1299. [31]	Perforated stomach lesions in rats. *J. Physiol. Pharmacol.* **2021**, *72(6)*. [33]
Primary abdominal compartment syndrome in rats. *Front. Pharmacol.* **2021**, *12*, 718147. [31]	Acute pancreatitis as vascular failure-induced severe peripheral and central syndrome in rats. *Biomedicines.* **2022**, *10*, 1299. [37]
Myocardial infarction induced by isoprenaline in rats. *Biomedicines*. **2022**, *10*, 265. [38]	Primary abdominal compartment syndrome in rats. *Front. Pharmacol.* **2021**, *12*, 718147. [31]
Over-dose lithium toxicity as an occlusive-like syndrome in rats. *Biomedicines* **2021**, *9*, 1506. [39]	Myocardial infarction induced by isoprenaline in rats. *Biomedicines*. **2022**, *10*, 265. [38]
Robert’s intragastric alcohol-induced gastric lesion model as an escalated general peripheral and central syndrome. *Biomedicines.* **2021**, *9*, 1300. [40]	Overdose lithium toxicity as an occlusive-like syndrome in rats. *Biomedicines* **2021**, *9*, 1506. [39]
	Robert’s intragastric alcohol-induced gastric lesion model as an escalated general peripheral and central syndrome. *Biomedicines.* **2021**, *9*, 1300. [40]
	Definitive and early spinal cord injury in rats. *Curr. Issues Mol. Biol.* **2022**, *44*, 1901–1927. [36]
	Amputation in rats treated with heparin, warfarin or aspirin. *Thromb. Res.* **2012**, *129*, 652–659. [58]
	Amputation in rats treated with heparin, warfarin, L-NAME and L-arginine. *PLoS ONE* **2015**, 10, e0123454. [59]
**Specifically maintained function of thrombocytes** **(aggregometry and thromboelastometry studies)**	
Intragastric application of aspirin, clopidogrel, cilostazol, and BPC 157 in rats: Platelet aggregation and blood clot. *Oxid. Med. Cell. Longev.* **2019**, *2019*, 9084643. [60]	

**Table 3:** Summarized presentation of the BPC 157 therapy effect on arrhythmias [66,67,68,69,70,71,72]. Note, in digitalis [66], potassium overdose [67], furosemide overdose [69], and bupivacaine [71] arrhythmias, BPC 157 might annihilate further worsening induced by NOS-blocker, L-NAME.

Noxious Procedure	BPC 157 Therapy Effects
Cumulative intravenous digitalis toxicity, methyldigoxin increment regimen (2.0/1.5/1.5/1.0 mg/kg at 15 min-intervals, total dose 6.0 mg/kg/45 min Advanced methyldigoxin toxicity (6.0 mg/kg i.v. bolus) [66].	BPC 157 (50 µg, 10 µg, 10 ng/kg) applied intravenously immediately before a methyldigoxin increment reduced the number of ventricular premature beats, prolonged the time before onset of ventricular tachycardia, reduced ventricular tachycardia and AV-block duration (µg-regimes) or reduced mainly the AV-block duration (ng-regimen). With the advanced methyldigoxin toxicity, BPC 157 applied at the 20th second of the grade 3 AV-block shortened AV-blocks, mitigated a further digitalis toxicity course. Ventricular tachycardias were either avoided (50 µg) or markedly reduced (10 µg, 10 ng). Fatal outcome was either avoided (50 µg), reduced (10 µg), or only delayed (10 ng).
Intraperitoneal KCl-solution application (9 mEq/kg). Promptly unrelenting hyperkalemia (>12 mmol/L), arrhythmias (and muscular weakness, hypertension, low pressure in lower esophageal and pyloric sphincter) with an ultimate and a regularly inevitable lethal outcome within 30 min. Intragastric KCl-solution application (27 mEq/kg)–(hyperkalemia 7 mmol/L): severe stomach mucosal lesions, sphincter failure, and peaked T waves HEK293 cells, hyperkalemic conditions (18.6 mM potassium concentrations), the effect on membrane potential, and depolarizations caused by hyperkalemic conditions [67].	Life-saving effect in severe hyperkalemia without affecting the extremely high level of potassium in blood. Given 30 min before KCl, all BPC 157 regimens regained sinus rhythm, had less prolongation of QRS, and had no asystolic pause. BPC 157 therapy, given 10 min after KCl-application, starts the rescue within 5–10 min, completely restoring normal sinus rhythm at 1 h. Likewise, other hyperkalemia disturbances (muscular weakness, hypertension, low sphincteric pressure) were also counteracted. Intragastric BPC 157 (10 ng, 10 μg) application, given 30 min before or 10 min after intragastric KCl, fully counteracted the severe stomach mucosal lesions, sphincter failure, and peaked T waves. In HEK293 cells, in hyperkalemic conditions (18.6 mM potassium concentrations), BPC 157 directly affects potassium conductance, counteracting the effect on membrane potential and depolarizations caused by hyperkalemic conditions.
Succinylcholine administration (1.0 mg/kg into the right anterior tibial muscle). Assessments were made at 3 and 30 min and one, three, five, and seven days after. The local paralytic effect Systemic muscle disability (and consequent muscle damage), Hyperkalemia, arrhythmias, and a rise in serum enzyme values [68]	BPC 157 successfully antagonized the depolarizing neuromuscular blocker effects of succinylcholine. BPC 157 (10 µg/kg, 10 ng/kg) (given intraperitoneally 30 min before or immediately after succinylcholine or per-orally in drinking water through 24 h until succinylcholine administration) mitigated both local and systemic disturbances. BPC 157 completely eliminated hyperkalemia and arrhythmias, markedly attenuated or eradicated behavioral agitation, muscle twitches, motionless resting, and completely eliminated post-succinylcholine hyperalgesia. BPC 157 immediately eliminated leg contractures and counteracted both edema and the decrease in muscle fibers in the diaphragm and injected/non-injected anterior tibial muscles.
Furosemide (100 mg/kg intraperitoneally)-diuresis-hypokalemia mortal course in rats Deadly hypokalemia (<2.7 mmol/L) Severe arrhythmias (i.e., polymorphic ventricular tachycardia („torsades de pointes“)) Lethal outcome occurred within 90–150 min [69]. Membrane voltages (*V*m) of HEK293 cells (the slow-whole cell patch clamp technique). Hypokalemic conditions (0.4 mM) cells hyperpolarized for −6.1 ± 1.1 mV [69].	Life-saving effect in severe hypokalemia without affecting the extremely low level of potassium in blood. With prophylactic application (BPC 157 given 15 min before furosemide), all BPC 157 regimens maintained sinus rhythm, had no ventricular premature beats, ventricular tachycardia, AV block, no prolongation of intervals and waves without reduction of amplitude. With delayed application (BPC 157 given 90 min after furosemide, with hypokalemia, 3rd grade AV block and/ or ventricular tachycardia being present), within 5–10 min, BPC 157 regimens normalized P, R, S, T waves, PR, RR, QRS, QT interval duration, R, S, T wave amplitude, total AV block, and terminated ventricular tachycardia. Likewise, BPC 157 eliminated skeletal muscle myoclonus. HEK293 cell in hypokaliemic conditions. In hypokalemic conditions (0.4 mM) cells hyperpolarized for −6.1 ± 1.1 mV. After first hypokalemic step, the solution 1 μM BPC-157 depolarized cells for 4.6 ± 1.6 mV. Repeating hypokalemic step in the presence of BPC 157, cells did not hyperpolarize (3.1 ± 1.6 mV). After washing BPC 157 from bath solution, under hypokalemic conditions, cells hyperpolarized again.
Bupivacaine (100 mg/kg IP) in rats Bradycardia, AV-block, ventricular ectopies, ventricular tachycardia, T-wave elevation, and asystole. All of the fatalities with T-wave elevation, high-degree AV-block, respiratory arrest, and asystole. Membrane voltages (Vm) in HEK293 cells. Bupivacaine (1 mM) alone caused depolarization of the cells [70].	BPC 157 as potential antidote for bupivacaine cardiotoxicity. Bradycardia, AV-block, ventricular ectopies, ventricular tachycardia, T-wave elevation, and asystole. All of the fatalities had developed T-wave elevation, high-degree AV-block, respiratory arrest, and asystole. These were largely counteracted by BPC 157 administration (50 µg/kg, 10 µg/kg, 10 ng/kg, or 10 pg/kg IP) given 30 min before or 1 min after the bupivacaine injection. When BPC 157 was given 6 min after bupivacaine administration and after the development of prolonged QRS intervals (20 ms), the fatal outcome was markedly postponed. Membrane voltages (Vm) in HEK293 cells demonstrated that in combination with BPC 157 (1 µm), the bupivacaine-induced depolarization was inhibited.
Lidocaine-induced local anesthesia via intraplantar application and axillary and spinal (L4-L5) intrathecal block, Lidocaine-induced arrhythmias, Lidocaine-induced convulsions, Lidocaine-induced HEK293 cell depolarization [71]	BPC 157 as antidote in its own against lidocaine and local anesthetics BPC 157 was applied immediately after lidocaine or 5 min before the application of lidocaine considerably ameliorated plantar presentation. BPC 157 medication considerably counteracted lidocaine-induced limb function failure. BPC 157 antagonized the lidocaine-induced bradycardia and eliminated tonic-clonic convulsions. Moreover, BPC 157 counteracted the lidocaine-induced depolarization of HEK293 cells.
During seven days, haloperidol (0.625, 6.25, 12.5, and 25.0 mg/kg ip), fluphenazine (0.5, 5.0 mg/kg ip), clozapine (1.0, 10.0 mg/kg ip), quetiapine (1.0, 10.0 mg/kg ip), sulpiride (1.6, 16.0 mg/kg ip), metoclopramide (2.5, 25.0 mg/kg ip) or (1.0, 10.0 mg/kg ip). Since very early, a prolonged QTc interval has been continually noted with haloperidol, fluphenazine, clozapine, olanzapine, quetiapine, sulpiride, and metoclopramide in rats as a common central effect not seen with domperidone [72].	To counteract neuroleptic- or prokinetic-induced prolongation of the QTc interval, rats were given a BPC 157 regimen once daily over seven days (10 μg, 10 ng/kg ip) immediately after each administration of haloperidol, fluphenazine, clozapine, quetiapine, sulpiride, metoclopramide or domperidone. Consistent counteraction appears with the stable gastric pentadecapeptide BPC 157. Thus, BPC 157 rapidly and permanently counteracts the QTc prolongation induced by neuroleptics and prokinetics.

## Data Availability

The data presented in this study are available on request from the corresponding author.
